# Improving genetic prediction by leveraging genetic correlations among human diseases and traits

**DOI:** 10.1038/s41467-017-02769-6

**Published:** 2018-03-07

**Authors:** Robert M. Maier, Zhihong Zhu, Sang Hong Lee, Maciej Trzaskowski, Douglas M. Ruderfer, Eli A. Stahl, Stephan Ripke, Naomi R. Wray, Jian Yang, Peter M. Visscher, Matthew R. Robinson

**Affiliations:** 1 Queensland Brain Institute, University of Queensland, Queensland, QLD 4072 Australia; 2 Stanley Center for Psychiatric Research, Broad Institute, Cambridge, MA 02142 USA; 3 Analytic and Translational Genetics Unit, Massachusetts General Hospital and Harvard Medical School, Boston, MA 02114 USA; 4 Institute for Molecular Bioscience, University of Queensland, Queensland, QLD 4072 Australia; 5 Centre for Population Health Research, School of Health Sciences and Sansom Institute of Health Research, University of South Australia, Adelaide, SA 5000 Australia; 6 Division of Genetic Medicine, Department of Medicine, Psychiatry and Biomedical Informatics, Vanderbilt Genetics Institute, Vanderbilt University Medical Center, Nashville, TN 37235 USA; 7 Institute for Genomics and Multiscale Biology, Icahn School of Medicine at Mount Sinai, New York, NY 10029 USA; 8 Department of Psychiatry and Psychotherapy, Charité, Campus Mitte, 10117 Berlin, Germany; 9 Department of Computational Biology, University of Lausanne, 1015 Lausanne, Switzerland; 10 Swiss Institute of Bioinformatics, CH-1015 Lausanne, Switzerland

## Abstract

Genomic prediction has the potential to contribute to precision medicine. However, to date, the utility of such predictors is limited due to low accuracy for most traits. Here theory and simulation study are used to demonstrate that widespread pleiotropy among phenotypes can be utilised to improve genomic risk prediction. We show how a genetic predictor can be created as a weighted index that combines published genome-wide association study (GWAS) summary statistics across many different traits. We apply this framework to predict risk of schizophrenia and bipolar disorder in the Psychiatric Genomics consortium data, finding substantial heterogeneity in prediction accuracy increases across cohorts. For six additional phenotypes in the UK Biobank data, we find increases in prediction accuracy ranging from 0.7% for height to 47% for type 2 diabetes, when using a multi-trait predictor that combines published summary statistics from multiple traits, as compared to a predictor based only on one trait.

## Abstract (web summary)

Genetic prediction of complex traits so far has limited accuracy because of insufficient understanding of the genetic risk. Here, Maier et al. develop an improved method for trait prediction that makes use of genetic correlations between traits and apply it to summary statistics of psychiatric diseases.

## Introduction

Personalised medicine, in which genetic testing is the basis for informing future health status and determining intervention, is effectively applied for a number of monogenic disorders^1^. For common complex disorders, which are those that are underlain by multiple genetic and environmental factors^2^, predictive genetic testing that can discriminate individuals who are most at risk is currently limited, mainly because much of the genetic variation remains poorly understood^3,4^. The potential of genetic risk prediction to (i) inform early interventions and (ii) aid diagnosis by identifying individuals with an increased genetic risk of disease could be improved substantially by increasing the accuracy of genetic risk predictors^5^. While genome-wide association studies (GWASs) of increased sample size will continue to unravel the role of genetic factors for complex diseases^6^, improved prediction models are also required to maximise the accuracy of a risk predictor.

GWASs use linear regression to independently estimate the effects of single-nucleotide polymorphisms (SNPs) across the genome, and commonly, these estimated SNP effects are then used to create a genetic risk predictor in independent samples^7–9^. However, this approach is not optimal because it either ignores linkage disequilibrium (LD) between markers or accounts for LD by discarding potentially informative SNPs^10^. Prediction accuracy of complex phenotypes can be improved by methods that jointly estimate the SNP associations to obtain SNP effect estimates with best linear unbiased predictor (BLUP) properties within a linear mixed model (LMM) approach, a model termed genomic BLUP (GBLUP)^7,11,12^. A multi-trait extension of the LMM approach, yielding multivariate BLUP (MT-BLUP) predictors of the SNP effects, can further improve prediction accuracy when phenotypes are genetically correlated, because measurements on each trait provide information on the genetic values of the other correlated traits^13–16^. MT-BLUP has been shown to improve prediction accuracy for genetically correlated common psychiatric disorders when combining individual-level data across independent data sets^16,17^. However, the application of MT-BLUP to complex common disorders is limited as combining individual-level genotype-phenotype data across case–control studies of all complex diseases is generally not feasible due to data protection concerns and restrictions on data sharing.

Here we overcome this limitation by developing a framework that combines publically available GWAS summary statistics across multiple studies of different traits together in a weighted index to generate approximate multi-trait summary statistic BLUP (wMT-SBLUP) predictors (Supplementary Table 1). We show through theory and simulation study that MT-BLUP predictors, which traditionally require individual-level phenotype–genotype data for all traits, can be approximated accurately by wMT-SBLUP predictors in a computationally efficient manner using only summary statistic data and an independent genomic reference sample. We also show how multi-trait summary statistic predictors can be created directly from GWAS summary statistics (wMT-GWAS) or from predictors obtained using the software LDPred^18^ that extends a single-trait summary statistic BLUP model (SBLUP) by assuming that marker effects come from a mixture of distributions. We apply our approach to multiple phenotypes in the Psychiatric Genomics Consortium (PGC) to compare summary statistic approaches to direct estimation on individual-level data. We further apply our approach to summary statistics of several other phenotypes to create predictors that we evaluate using the UK Biobank data. We show that, for most traits, our multi-trait predictors improve prediction accuracy as compared to a single-trait predictors.

## Results

### Overview of the approach

**Fig. 1:**
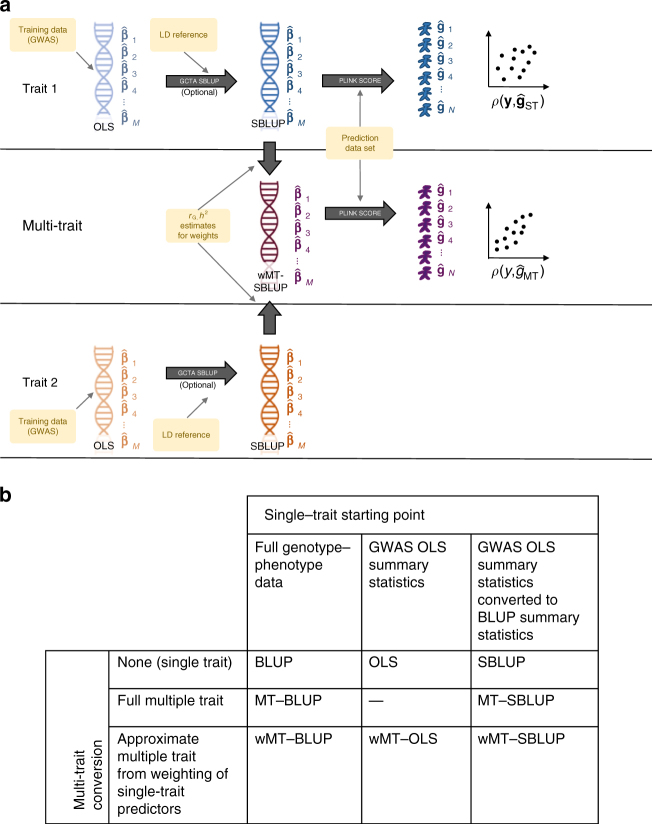
Schematic of the methods. **a** Data and programs used to create predictors. **b** Terminology to refer to different types of predictors. OLS, ordinary least squares. The most common GWAS methodology to estimate SNP effects is to estimate the effect sizes of one SNP at a time using linear regression. BLUP, best linear unbiased prediction. SNP effects are estimated simultaneously for all SNPs. The estimates depend on the other SNPs included in the analysis, since the contribution from correlated SNPs will be shared between them

Standard GWAS summary statistics are ordinary least squares (OLS) estimates of the SNP effects and do not have optimal properties for prediction^11^. Even when LMM association analysis is used, the estimated SNP effects still represent marginal effects and not effects conditional on other SNPs, which is what is desirable for prediction^19^. Previous studies have shown how OLS summary statistics can be reanalysed in a mixed model framework to produce approximate BLUP predictors (summary statistic BLUP: SBLUP, implemented in the most recent release of GCTA)^18,20,21^ or approximate mixture model predictors (LDPred). We first extend the SBLUP approach to a multi-trait framework (MT-SBLUP) and find a computational limitation associated with the inversion of a SNP-by-SNP-by-trait matrix. To overcome this, we then derive theory to show how single-trait predictors with BLUP properties can be combined together in a weighted index to generate predictors with equivalent properties to those gained from a MT-BLUP analysis (Fig. 1).

Consider two genetically correlated traits for which we have individual-level genetic predictors with BLUP properties. For each individual, *i*, and focal trait of interest, *f*, we have a genetic prediction $\left( {\widehat {\bf{g}}_{{\mathrm{BLUP}}_{i,k}}} \right)$ for each trait, *k*, that we can combine together using the index weights, *w*_*i*,*k*_, for each $\widehat {\bf{g}}_{{\mathrm{BLUP}}_{i,k}}$ effect to produce a weighted multi-trait BLUP genetic predictor:

1 $$\widehat {\bf{g}}_{{\mathrm{wMT}} - {\mathrm{BLUP}}_{i,f}} = \mathop {\sum }\limits_k w_{i,k}{\hat{\bf g}}_{{\mathrm{BLUP}}_{i,k}} = {\bf{w}}_i\prime \widehat {\bf{g}}_{{\mathrm{SBLUP}}_i}$$

In the Methods section, we show that the optimal index weights can be calculated as:

2 $${w} = \left[ {\begin{array}{*{20}{c}} {w_1} \\ {w_2} \end{array}} \right] = \left[ {\begin{array}{*{20}{c}} {R_1^2} & {\frac{{r_{\mathrm{G}}R_1^2R_2^2}}{{\sqrt {h_1^2h_2^2} }}} \\ {\frac{{r_{\mathrm{G}}R_1^2R_2^2}}{{\sqrt {h_1^2h_2^2} }}} & {R_2^2} \end{array}} \right]^{ - 1}\left[ {\begin{array}{*{20}{c}} {R_1^2} \\ {r_{\mathrm{G}}\sqrt {\frac{{h_1^2}}{{h_2^2}}} R_2^2} \end{array}} \right]$$

where $h_k^2$ is the SNP heritability of trait *k* (proportion of phenotypic variance explained by genome-wide SNPs), *r*_G_ is the genetic correlation between trait *k* and the focal trait and $R_k^2$ is the expected squared correlation between a phenotype and a BLUP predictor, calculated as:

3 $$R_k^2 = \frac{{h_k^2}}{{1 + M_{{\mathrm{eff}}}\frac{{1 - R_k^2}}{{N_kh_k^2}}}}$$

where *M*_eff_ is the effective number of chromosome segments and *N*_*k*_ is the sample size of trait *k*. These weights will ensure that the contribution of each added trait is approximately proportional to the square root of its sample size, its SNP heritability and its genetic correlation with the focal trait (trait 1), while accounting for different variances of single-trait BLUP predictors.

Both $h_k^2$ and *r*_G_ can be estimated from GWAS summary statistics using LD score regression^22,23^. Following^20^, individual-level genetic predictors with BLUP properties can also be obtained from GWAS summary statistics ($\widehat {\bf{g}}_{{\mathrm{SBLUP}}_k}$, where SBLUP represents summary statistic approximate BLUP). Therefore, for any given trait, genetic predictors with BLUP properties $\left( {\widehat {\bf{g}}_{{\mathrm{SBLUP}}_k}} \right)$ can be created from GWAS summary statistics and these can then be placed in a weighted index to produce approximate multi-trait summary statistic BLUP (wMT-SBLUP) predictors, using only LD score regression and an independent reference sample. This approach, provided in the freely available software SMTPred (see Code availability section), approximates MT-BLUP predictors without the need for individual-level phenotype–genotype data for all traits, enabling prediction accuracy to be improved by fully utilising all of the publically available GWAS summary statistic data. We also show how weighted indices can be calculated for GWAS summary statistics (wMT-GWAS) or from predictors obtained using the software LDPred^18^ (wMT-LDPred), therefore depending upon the genetic architecture of the trait approximate multi-trait summary statistics can be created to maximise genomic prediction accuracy.

### Simulation study

**Fig. 2:**
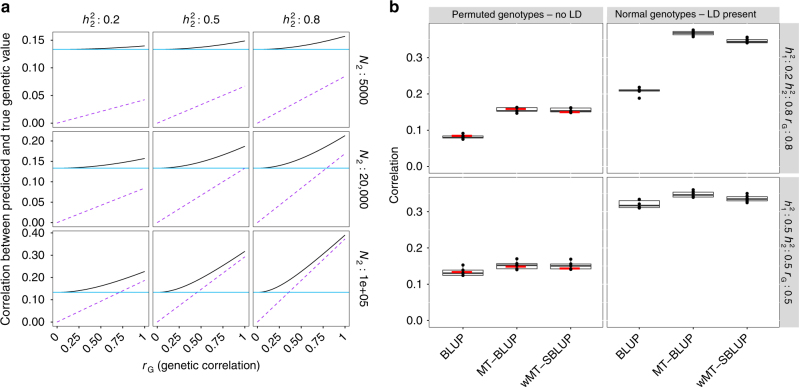
Improving prediction accuracy using information from multiple traits. **a** Expected gain from multi-trait vs cross-trait predictors as a function of *r*_G_. Two traits are considered. The first trait has a sample size of 20,000 and a SNP heritability of 0.5. The sample size and SNP heritability of the second trait vary between panels. The blue line shows the expected prediction accuracy of a single-trait predictor. The black line shows the expected prediction accuracy of a multi-trait predictor. The purple line shows the expected prediction accuracy of a cross-trait predictor (using only trait 2 to predict trait 1). The advantage of a multi-trait predictor over a cross-trait predictor decreases with increasing *r*_G_, *h*^2^, and sample size of the second trait. **b** Simulation results. Prediction accuracy is shown as correlation between simulated genetic value and predicted phenotype of individuals. Genotypes from European individuals in the GERA cohort were used for simulation. Boxplots show results across six replicates. In the left panels, the LD structure was removed by permuting dosage values for each SNP across all individuals. In the right panels, the original genotypes were used for simulation. Expected prediction accuracies were derived for the case of unlinked genotypes and are shown as red horizontal bars. In each section, the prediction accuracy of three predictors is shown: (1) single trait BLUP, (2) multi-trait BLUP (MT-BLUP), and (3) weighted approximate BLUP (summary statistic-based multi-trait predictor: wMT-SBLUP). Simulation in genotypes without LD results in prediction accuracies, which conform to expectations. In the presence of LD, the expected prediction accuracy depends very much on the choice of *M*_eff_

We first conducted a simulation study using observed SNP genotype data to confirm the expectations from our theory. We show through theory (see Methods section) that a wMT-SBLUP genetic predictor has the same expected prediction accuracy as one created from a multivariate mixed-effects model (multi-trait BLUP: MT-BLUP) if the linkage disequilibrium among SNP markers in the individual-level analysis is well approximated by a reference genotype panel (see Methods section). We demonstrate that a wMT-SBLUP predictor increases prediction accuracy over a single-trait predictor, with the magnitude of increase being proportional to the ratio of the SNP heritability of the added traits relative to that of the predicted trait, the sample size of the added traits relative to that of the predicted trait and the genetic correlation between the added traits and the predicted trait (Fig. 2, Supplementary Figs. 1 and 2). We also demonstrate how genetic predictors generated by LDPred^18^ can be combined in an approximate multi-trait weighting (Supplementary Fig. 3).

We also provide a theoretical expectation for the loss in prediction accuracy that occurs when using an independent reference sample to compute SBLUP effects compared to a predictor based on BLUP effects (see Methods section), and we detail the loss of prediction accuracy in our simulation study (Fig. 2b, Supplementary Figs. 1 and 4).

### Application to psychiatric disorders

**Fig. 3:**
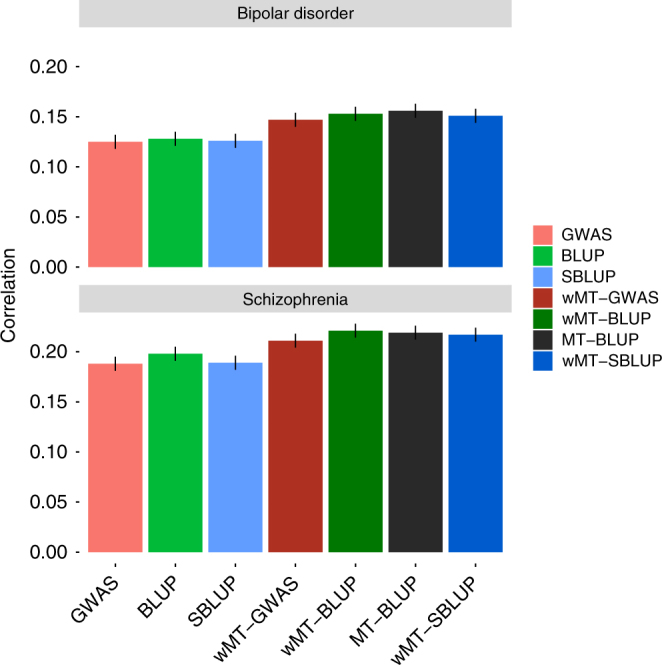
Prediction accuracy for schizophrenia and bipolar disorder from several single-trait and multi-trait predictors. Prediction accuracy of seven different types of predictors using PGC1 schizophrenia and bipolar disorder data. Single-trait predictor (lighter colours) are on the left, multi-trait predictors (darker colours) are on the right. Black error bars indicate correlation coefficient standard errors, calculated as ${\mathrm{se}}_r = \sqrt {\frac{{1 - r^2}}{{n - 2}}}$

We then applied our approach to the PGC schizophrenia^24,25^ and bipolar data, two psychiatric disorders known to have a high genetic correlation^26^. The availability of combined individual-level data for both disorders enabled a direct comparison of the MT-BLUP^16^ and wMT-SBLUP approaches. We calculated all predictors for the previously used^16^ PGC wave 1 (PGC1) data sets^24^ and compared the prediction accuracy (correlation between predicted values and phenotypes adjusted for sex, cohort and the first 20 principal components) across diseases and approaches. We find comparable but slightly lower accuracies in the wMT-SBLUP predictors as compared to the MT-BLUP predictors (0.151 vs 0.156 in bipolar disorder and 0.217 vs 0.219 in schizophrenia) and an increase in prediction accuracy as compared to the single-trait (BLUP) predictors (0.128 in bipolar disorder, 0.198 in schizophrenia) (Fig. 3). Our results demonstrate that creating SBLUP genetic predictors using an independent LD reference sample and combining these in a weighted sum results in prediction accuracy comparable to a full MT-BLUP prediction for common complex disease traits, at a much lower computational burden.

We then applied our approach to the larger PGC wave 2 (PGC2) data sets for schizophrenia^25^ and bipolar disorder (see Methods section), which included the PGC1 data. To test whether the addition of more cohorts improved prediction accuracy, we estimate wMT-SBLUP predictors in the PGC2 data. Having shown the resemblance of wMT-SBLUP and MT-BLUP by theory, simulation and in the PGC1 data, we refrained from running a MT-BLUP model in the PGC2 data to avoid the computational burden of analysing the combined schizophrenia bipolar data set. For schizophrenia, there were 36 cohorts (26,412 cases and 32,440 controls in total) and for bipolar disorder there were 23 cohorts (18,865 cases and 30,460 controls in total). We conducted a cohort-wise leave-one-out cross-validation approach to examine variation in prediction accuracy across cohorts.

For schizophrenia, we find that prediction accuracy increases in 20 of the 36 cohorts of the PGC2 data when using a wMT-SBLUP predictor as compared to a SBLUP predictor (Supplementary Fig. 5). However, the median correlation (0.300 with an SBLUP predictor, and 0.304 with a wMT-SBLUP predictor) and mean correlation (0.295 with a SBLUP predictor and 0.294 with a wMT-SBLUP predictor) across the 36 PGC2 cohorts did not improve with a wMT-BLUP predictor. For bipolar disorder, we find an improvement of the wMT-SBLUP predictor over the SBLUP predictor in 17 out of the 23 cohorts (Supplementary Fig. 6), with a mean correlation increase from 0.212 to 0.229 and a median correlation increase from 0.210 to 0.225. To evaluate whether this is because the weights we used for schizophrenia and bipolar disorder do not represent the mixing proportions that lead to the highest accuracy in this data set or whether other factors explain the variable results across cohorts, we created multi-trait predictors using not only weights calculated from Eq. (17) but also weights corresponding to any other mixing proportion of the two disorders (Supplementary Figs. 5, 6 and 7). This demonstrates (i) that our calculated weights are very close to the empirically optimal weights when averaged across cohorts (Supplementary Fig. 7), (ii) that there is substantial heterogeneity across cohorts as shown by the variable prediction accuracies of single-trait and cross-trait predictors across cohorts, which is supported by previous studies^25^, and (iii) that, for some test set cohorts, there is no mixing proportion that will lead to a multi-trait predictor which outperforms a single-trait predictor. The larger gain in accuracy that results from supplementing a bipolar disorder predictor with schizophrenia data compared to supplementing a schizophrenia predictor with bipolar disorder data is consistent with greater power of the schizophrenia discovery sample. We find that for both single-trait and multi-trait predictors the SBLUP predictors outperform the OLS predictors in almost all cohorts (Supplementary Figs. 5 and 6).

### Application to traits recorded in a large population study

**Fig. 4:**
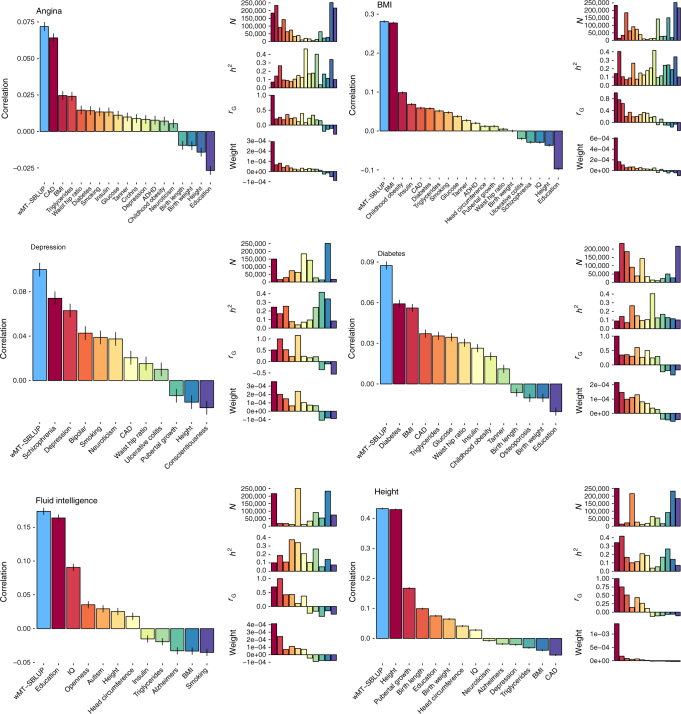
Prediction accuracy for single-trait and multi-trait predictors in UK Biobank traits. Prediction accuracy for six traits in the UK Biobank for multi-trait predictors (light blue bars, wMT-SBLUP) and single-trait predictors (colourful bars on the right, SBLUP). Black bars show the correlation coefficient standard error. The multi-trait predictors for each trait are composed of all traits for which colourful bars are shown (*r*_G_*p*-value < 0.05). Smaller bars on the right show, from top to bottom, sample size, SNP heritability, *r*_G_, and weights (given by Eq. (15)) for each trait

In principle, any number of traits can be combined into a multi-trait predictor at almost no computational cost. We therefore extended our approach to create wMT-SBLUP predictors from 34 phenotypes for which we could access summary statistics. In order to calculate wMT-SBLUP weights, we used LD score regression to estimate SNP heritability and genetic correlations of the 34 summary statistics traits. The results are mostly in line with previous reports^23^ (Supplementary Fig. 8, Supplementary Data 1). As test set, we used 112,338 individuals in the UK Biobank data. We matched 6 of the 34 discovery traits to traits in the UK Biobank (Supplementary Table 1) and created wMT-SBLUP predictors. For the wMT-SBLUP predictor of each focal trait, we included predictor traits with genetic correlation *p*-value < 0.05. For all traits, wMT-SBLUP genetic predictors were more accurate than any single-trait (SBLUP) predictor (Fig. 4). wMT-SBLUP predictors generally improved prediction accuracy over single-trait GWAS OLS predictors (Supplementary Fig. 9) and were similar to wMT-LDPred predictors (Supplementary Figs. 10 and 11.) We observe the largest increases in accuracy for Type 2 diabetes (47.8%) and depression (34.8%). Accuracy for height (0.7%) and body mass index (BMI) (1.4%) increase only marginally. As shown in our theory and simulation study, the magnitude of increase in prediction accuracy of a wMT-SBLUP predictor over a single-trait SBLUP predictor depends upon the prediction accuracies of all the traits included in the index and the genetic correlation among phenotypes. As GWAS sample sizes increase and genomic predictors increase in accuracy, a wMT-SBLUP approach will likely become increasingly beneficial.

## Discussion

In summary, we demonstrate that multivariate predictors derived from GWAS summary statistics can increase prediction accuracy in a wide range of traits. This approach has particular utility in risk prediction of traits for which it is hard to generate large sample sizes for GWAS, as SNP heritability and sample size are the two factors that determine prediction accuracy of a polygenic trait, when using a single-trait predictor. The increase in prediction accuracy of a multi-trait over a standard single-trait genetic predictor is therefore greatest when the additional traits included in the predictor have higher SNP heritability and sample size than the trait to be predicted, as well as a high genetic correlation with the trait to be predicted. We show how genetic predictors from GWAS OLS effects, LDPred effects or SBLUP effects can be combined, yielding an approach that is general across different phenotypes.

Special consideration should be given to the risk of sample overlap between the summary statistics data used to create the predictor and the prediction target. Sample overlap will lead to inflated estimates of accuracy, and while here we were able to take steps to avoid individuals being recorded across multiple data sets, further work is required to negate these effects within this framework. In principle, assuming perfect homogeneity between training and test set and perfect estimates of SNP heritability and genetic correlation, there is no limit to the number of traits that can be combined using our approach. In practice, however, there will be little benefit of combining traits with low genetic correlation, as they will not influence the predictor much. Some added traits might even reduce accuracy, if the genetic correlation is not estimated accurately. The focus of our analysis was the prediction of genetic risk and we aimed to provide a fast, computationally efficient, general framework for genomic prediction. This sets it apart from other multi-trait approaches like phenome-wide association studies, which focus on the effects of individual SNPs on multiple phenotypes. We note, however, that a multi-trait testing approach can in principle also be used to increase the power to identify loci associated with specific traits as demonstrated in the recently developed MTAG method^27^. Another potential caveat of our analysis is that prediction accuracy increases for a focal trait may come from the addition of traits that are standardly measured on patients, and improved frameworks are required to identify marker effects conditionally on known health risk factors. Despite these limitations, current evidence suggests that genetic correlations among phenotypes are pervasive^23^, sample sizes of GWAS are increasing^6^ and public availability of genome-wide summary statistics is becoming the norm^28^, meaning that genomic prediction of complex common disease will continually improve especially when predictors of multiple phenotypes are integrated across studies within this framework.

## Methods

### General model

We consider a general linear mixed model:

4 $${\bf{y}} = {\bf{Wb}} + {\bf{\epsilon }}$$

where **y** is the phenotype, **W** a matrix of SNP genotypes, where values are standardised to give the *ij*^th^ element as: $w_{ij} = \left( {x_{ij} - 2p_j} \right){\mathrm{/}}\sqrt {2p_j\left( {1 - p_j} \right)}$, with *x*_*ij*_ the number of minor alleles (0, 1 or 2) for the *i*^th^ individual at the *j*^th^ SNP and *p*_*j*_ the minor allele frequency. **b** are the genetic effects for each SNP, and ${\bf{\epsilon }}$ the residual error. The dimensions of **y**, **W**, **b** and ${\bf{\epsilon }}$ are dependent upon the number of phenotypes, *k*, the number of SNP markers, *M*, and the number of individuals, *N*, and are described in the sections below. We denote the distributional properties var(**b**) = **B**, var(${\bf{\epsilon }}$) = **R** and var(**y**) = **WBW**′ + **R**.

For human complex diseases and quantitative phenotypes, GWASs have typically estimated the solutions for **b** of Eq. (1) one SNP at a time using OLS regression^29^ as:

5 $$\widehat {\bf{b}}_{{\mathrm{OLS}}} = {\mathrm{diag}}\left[ {{{{\bf W}{\prime}{\bf W}}}} \right]^{ - 1}{{{\bf W}{\prime}{\bf y}}}$$

where diag[**W**′**W**] has diagonal elements $w_j\prime w_j$ and off-diagonal elements of zero. However, by analysing one SNP at a time, GWAS effect size estimates do not account for the covariance structure among SNPs and they are not unbiased in the sense that ${\mathit{E}}\left[ {{\bf{b}}|{\hat{\bf b}}} \right] = {\hat{\bf b}}$^12^. BLUP of the SNP effects have the property ${\it{E}}\left[ {{\bf{b}}|{\hat{\bf b}}} \right] = {\hat{\bf b}}$, are used in genomic prediction in animal and plant breeding^30^ and more recently in human medical genetics, yielding improved prediction accuracy for a number of traits over genetic predictors created from OLS SNP estimates^16,17^. In a general form, BLUP solutions for **b** of Eq. (1) can be written using Henderson’s mixed model equations^31^ as:

6 $$\widehat {\bf{b}}_{{\mathrm{BLUP}}} = \left[ {{{{\bf W}{\prime}{\bf R}}}^{ - {\mathrm{1}}}{\bf{W}}{{ + }}{\bf{B}}^{ - 1}} \right]^{ - 1}{{{\bf W}{\prime}{\bf R}}}^{ - 1}{\bf{y}}$$

and if **R** is diagonal, then Eq. (6) can be reduced to:

7 $$\widehat {\bf{b}}_{{\mathrm{BLUP}}} = \left[ {{{{\bf W}{\prime}{\bf W}}} + {\bf{B}}^{ - 1}{\bf{R}}} \right]^{ - 1}{{{\bf W}{\prime}{\bf y}}}$$

Below, we describe how Eqs. (6) and (7) can be used to estimate BLUP SNP effects for a single trait and for multiple traits jointly from individual-level phenotype–genotype data. We then show how Eqs. (6) and (7) can be approximated to obtain BLUP SNP effects for single and multiple traits in the absence of individual-level data from publically available GWAS summary statistics and an independent reference sample.

### Estimation of BLUP SNP effects for a single trait

For a univariate analysis of trait *k*, **y** of Eq. (4) is a column vector of length *N* × 1 and **W** has dimension *N* × *M*. Assuming **b** is an *M* × 1 vector of random SNP effects for trait *k*, with distribution ${\bf{b}}\sim N\left( {0,{\bf{I}}_M\sigma _{b_k}^2} \right)$, then ${\bf{B}} = {\bf{I}}_M\sigma _{b_k}^2$ with **I**_*M*_ is an identity matrix of dimension *M*. ${\bf{\epsilon }}$ of Eq. (1) is a column vector of independent residual effects, with distribution ${\bf{\epsilon }}\sim N\left( {0,{\bf{I}}_N\sigma _{{\it{\epsilon }}_k}^2} \right)$, giving ${\bf{R}} = {\bf{I}}_N\sigma _{\epsilon _k}^2$, with **I**_*N*_ an identity matrix of dimension *N*. Substituting these expressions into Eq. (6) means that Eq. (7) can then be written as:

8 $$\widehat {\bf{b}}_{{\mathrm{BLUP}}_k} = \left[ {{\bf{W}}_k\prime {\bf{W}}_k + {\bf{I}}_M\lambda _k} \right]^{ - 1}{\bf{W}}_k\prime {\bf{y}}_k$$

with $$\lambda _k = \sigma _{\epsilon _k}^2{\mathrm{/}}\sigma _{{\it{b}}_k}^2$$.

### Joint estimation of BLUP SNP effects for multiple traits

Phenotypic measurements of a trait can be informative for the genetic values of other traits, if the traits are genetically correlated with one another^14,15,32^. Recent studies have shown that prediction accuracy of common complex disease can be improved by estimating SNP effects for multiple traits jointly within a multivariate mixed-effects model^16,17^.

If *k* traits are measured on different individuals, with *N*_*k*_ observations for trait *k*, the elements of Eq. (4) become: $${{\bf y}^ \prime} = [{\bf y}\prime_{\bf 1}...{{\bf y}^ \prime_{k}}], {\bf{W}} = \left[ {\begin{array}{*{20}{c}} {{{\bf W}}_1} & 0 & 0 \\ 0 & \ddots & 0 \\ 0 & 0 & {{\bf{W}}_k} \end{array}} \right]$$, and **R** = diag**[R**_*k*_] = diag$\left[ {{\bf{I}}_{N_k}\sigma _{\epsilon _k}^2} \right]$, a diagonal matrix of length $N = \mathop {\sum }\limits_k N_k$. ${\bf{B}} = {\bf{\Sigma }}_b \otimes {\bf{I}}_M$, where **Σ**_*b*_ is a *k* × *k* matrix, with diagonal elements $\sigma _{b_k}^2$ and off-diagonal elements the covariances of SNP effects between traits *k* and *l*, $\sigma _{b_{k,l}}$. For Kronecker products, ${\bf{B}}^{ - 1} = {\bf{\Sigma }}_b^{ - 1} \otimes {\bf{I}}_M$ and substituting these expressions directly into Eq. (6) means that multi-trait BLUP solutions for **b** can be obtained in Eq. (7) as:

9 $$\widehat {\bf{b}}_{{\mathrm{MT}} - {\mathrm{BLUP}}} = \left[ {{{{\bf W}{\prime}{\bf W}}} + {\bf{\Sigma }}_\epsilon {\bf{\Sigma }}_b^{ - 1} \otimes {\bf{I}}_M} \right]^{ - 1}{{{\bf W}{\prime}{\bf y}}}$$

with ${\bf{\Sigma }}_\epsilon = {\mathrm{diag}}\left[ {\sigma _{\epsilon _k}^2} \right]$, a diagonal *k* × *k* matrix. For a two-trait example, Eq. (9) expands to:

10 $$\begin{array}{*{20}{l}} {\widehat {\bf{b}}_{\mathrm{MT-BLUP}}} \hfill & = \hfill & {\left[ {\left[ {\begin{array}{*{20}{c}} {{\bf{W}}_1\prime {\bf{W}}_1} & 0 \\ 0 & {{\bf{W}}_2\prime {\bf{W}}_2} \end{array}} \right]} \right.} \hfill \\ {} \hfill & + \hfill & {\left. {\left[ {\begin{array}{*{20}{c}} {{\bf{I}}_M\sigma _{\epsilon _1}^2} & 0 \\ 0 & {{\bf{I}}_M\sigma _{\epsilon _2}^2} \end{array}} \right]\left[ {\begin{array}{*{20}{c}} {{\bf{I}}_M\sigma _{b_1}^2} & {{\bf{I}}_M\sigma _{b_{1,2}}} \\ {{\bf{I}}_M\sigma _{b_{2,1}}} & {{\bf{I}}_M\sigma _{b_2}^2} \end{array}} \right]^{ - 1}} \right]^{ - 1}} \hfill \\ {} \hfill & {} \hfill & {\left[ {\begin{array}{*{20}{c}} {{\bf{W}}_1} & 0 \\ 0 & {{\bf{W}}_2} \end{array}} \right]\prime \left[ {\begin{array}{*{20}{c}} {{\bf{y}}_1} \\ {{\bf{y}}_2} \end{array}} \right]} \hfill \end{array}$$

### Multi-trait BLUP SNP effects from summary statistics

Estimating SNP effects for multiple traits jointly in Eq. (9) requires individual-level genotype and phenotype data across a range of common complex diseases and quantitative phenotypes, which are not readily available in human medical genetics due to privacy concerns and data sharing restrictions. Additionally, Eq. (9) requires a series of computationally intensive *M* × *k* equations to be solved. However, these issues can be overcome by approximating Eq. (9) using publically available GWAS summary statistic data and an independent genomic reference sample.

Single-trait approximate BLUP SNP effects can be obtained from GWAS summary statistics (SBLUP: summary statistic approximate BLUP) by replacing ${\bf{W}}_k\prime {\bf{W}}_k$ and ${\bf{W}}_k\prime {\bf{y}}_k$ of Eq. (8) by their expectation, which are ${\Bbb E}\left[ {{\bf{W}}_k\prime {\bf{W}}_k} \right] = N_k{\bf{L}}$ and ${\Bbb E}\left[ {{\bf{W}}_k\prime {\bf{y}}_k} \right] = N_k\widehat {\bf{b}}_{{\mathrm{OLS}}_k}$, respectively, where **L** is an *M* × *M* scaled SNP LD correlation matrix estimated from a reference SNP data set and $\widehat {\bf{b}}_{{\mathrm{OLS}}_k}$ are obtained from publically available GWAS summary statistics^20^. GWAS summary statistics report effect estimates of SNPs on an unstandardised scale and not $\widehat {\bf{b}}_{{\mathrm{OLS}}}$ as it is defined here. To obtain $\widehat {\bf{b}}_{{\mathrm{OLS}}}$ from GWAS summary statistics, the effect of each SNP must be multiplied by the standard deviation of each SNP: $\widehat {\bf{b}}_{{\mathrm{OLS}}_{\mathrm{j}}}$ = $\widehat {\bf{b}}_{{\mathrm{OLS}} - {\mathrm{UNSCALED}}_{\mathrm{j}}} \times \sqrt {2p_j\left( {1 - p_j} \right)}$. Equation (8) can then be written as:

11 $$\begin{array}{*{20}{l}} {\widehat {\bf{b}}_{{\mathrm{SBLUP}}_k}} \hfill & = \hfill & {\left[ {N_k{\bf{L}} + {\bf{I}}_M\lambda _k} \right]^{ - 1}N_k\widehat {\bf{b}}_{{\mathrm{OLS}}_k}} \hfill \\ {} \hfill & = \hfill & {\left[ {{\bf{L}} + {\bf{I}}_M\lambda _k{\mathrm{/}}N_k} \right]^{ - 1}\widehat {\bf{b}}_{{\mathrm{OLS}}_k}} \hfill \end{array}$$

The shrinkage parameter is $\lambda _k = \sigma _{{\it{\epsilon }}_k}^2{\mathrm{/}}\sigma _{b_k}^2$ = $M\sigma _{{\it{\epsilon }}_k}^2{\mathrm{/}}h_{{\rm SNP}_k}^2$ = $M\left( {1 - h_{{\rm SNP}_k}^2} \right){\mathrm{/}}h_{{\rm SNP}_k}^2$, under the assumption of phenotypic variance of 1 that makes the proportion of phenotypic variance of trait *k* attributable to the SNPs $h_{{\rm SNP}_k}^2 = M\sigma _{b_k}^2$.

This approach was implemented in ref. ^21^ and is similar to the LDpred model presented by Vilhjálmsson et al.^18^ but with a few differences. The first is that it only considers the infinitesimal case, where all SNPs are considered to be causal and their effect sizes follow a normal distribution. This corresponds to the LDpred-Inf model. The second difference is that the shrinkage parameter of Vilhjálmsson et al.^18^ is $\lambda _k = M{\mathrm{/}}h_{{\mathrm{SNP}}_k}^2$ as they assume that the error variance is 1 rather than $1 - h_{{\mathrm{SNP}}_k}^2$ in our implementation. The third difference is that in the LDpred-Inf model, Vilhjálmsson et al.^18^ calculate BLUP effects for blocks of a certain number of SNPs following a tiling window approach giving a block diagonal structure to **L**, whereas our implementation within the software GCTA (see URLs) follows a sliding window approach giving a banded diagonal to **L**. Assuming an error variance of $1 - h_{{\mathrm{SNP}}_k}^2$ is more appropriate because cumulatively the SNP markers explain $h_{{\mathrm{SNP}}_k}^2$ of the phenotypic variance. In both implementations, a window is used to capture the LD around SNP markers in order to avoid the large computational costs of inverting a dense *M* dimensional SNP LD matrix, with only little loss of information (see below).

For multiple phenotypes, the elements of Eq. (11) become: $\widehat {\bf{b}}_{{{{\rm OLS}{\prime}}}} = \left[ {\widehat {\bf{b}}_{{\mathrm{OLS}}_1{\prime} } \ldots \widehat {\bf{b}}_{{\mathrm{OLS}}_k{\prime} }} \right]$ and **N** = $$\left[ {\begin{array}{*{20}{c}} {{\bf{N}}_1} & 0 & 0 \\ 0 & \ddots & 0 \\ 0 & 0 & {{\bf{N}}_k} \end{array}} \right]$$, meaning that Eq. (11) can be extended as:

12 $$\widehat {\bf{b}}_{{\mathrm{MT}} - {\mathrm{SBLUP}}} = \left[ {{\bf{I}}_k \otimes {\bf{L}} + {\bf{\Sigma }}_\epsilon {\bf{\Sigma }}_b^{ - 1}{\bf{N}}^{ - 1} \otimes {\bf{I}}_M} \right]^{ - 1}\widehat {\bf{b}}_{{\mathrm{OLS}}}$$

Equation (12) approximates Eq. (9) using only publically available GWAS summary statistic data and an independent genomic reference sample. However, there remains the large computational cost associated with the inversion of the non-diagonal matrix $\left[ {{\bf{I}}_k \otimes {\bf{L}} + {\bf{\Sigma }}_\epsilon {\bf{\Sigma }}_b^{ - 1}{\bf{N}}^{ - 1} \otimes {\bf{I}}_M} \right]$.

### Index weighted multi-trait BLUP SNP effects

An alternative to Eq. (12), is to obtain *k* $\widehat {\bf{b}}_{{\mathrm{MT}} - {\mathrm{SBLUP}}}$ effects by combining together *k* single-trait $\widehat {\bf{b}}_{{\mathrm{SBLUP}}}$ estimates of Eq. (11), using an optimal index weighting for each trait. The index weighting to derive $\widehat {\bf{b}}_{{\mathrm{MT}} - {\mathrm{SBLUP}}}$ from $\widehat {\bf{b}}_{{\mathrm{SBLUP}}}$ estimates is identical to the index weighting to derive $\widehat {\bf{b}}_{{\mathrm{MT}} - {\mathrm{BLUP}}}$ from $\widehat {\bf{b}}_{{\mathrm{BLUP}}}$ estimates.

For SNP *j* and focal trait *f*, we have $\widehat {\bf{b}}_{{\mathrm{SBLUP}}}$ values for *k* traits, and we wish to obtain the index weights, *w*_*j*,*k*_, for each $\widehat {\bf{b}}_{{\mathrm{SBLUP}}_{j,k}}$ effect as:

13 $$\widehat {\bf{b}}_{\mathrm{wMT-SBLUP}_{j,f}} = \mathop {\sum} \limits_{k} {\bf{w}}_{{\mathrm{SBLUP}},j,k}\widehat{\bf{b}}_{{\mathrm{SBLUP}}_{j,k}} = {\bf{w}}_{{\mathrm{SBLUP}},j}{\prime}\widehat {\bf{b}}_{{\mathrm{SBLUP}}_j}$$

In animal and plant breeding, selection indices have been developed, which combine many single-trait BLUP predictors of an individual’s genetic value together in an index weighting to optimise the selection of individuals with the most favourable multi-trait phenotype for breeding programs^33–36^. Utilising a selection index approach, the solution for **w**_SBLUP_ of Eq. (13) can be obtained as:

14 $${\bf{w}}_{{\mathrm{SBLUP}}}={\bf{V}}_{{\mathrm{SBLUP}}}^{-1}{\bf{C}}_{{\mathrm{SBLUP}}}$$

where **C**_SBLUP_ a *k* × 1 column vector of the covariance of the $\widehat {\bf{b}}_{{\mathrm{SBLUP}}_k}$ values of the *k* traits, with the true genetic effects of the SNPs for the focal trait, and **V**_SBLUP_ a *k* × *k* variance–covariance matrix of the $\widehat {\bf{b}}_{{\mathrm{SBLUP}}}$ effects:

15 $$\begin{array}{*{20}{l}} {{\bf{w}}_{{\mathrm{SBLUP}}}} \hfill & = \hfill & {{\bf{V}}_{{\mathrm{SBLUP}}}^{ - 1}{\bf{C}}_{{\mathrm{SBLUP}}}} \hfill \\ {} \hfill & = \hfill & {\left[ {\begin{array}{*{20}{c}} {{\mathrm{var}}\left( {\widehat {\bf{b}}_{{\mathrm{SBLUP}}_1}} \right)} & \cdots & {{\mathrm{cov}}\left( {\widehat {\bf{b}}_{{\mathrm{SBLUP}}_1},\widehat {\bf{b}}_{{\mathrm{SBLUP}}_k}} \right)} \\ \vdots & \ddots & \vdots \\ {{\mathrm{cov}}\left( {\widehat {\bf{b}}_{{\mathrm{SBLUP}}_k},\widehat {\bf{b}}_{{\mathrm{SBLUP}}_1}} \right)} & \cdots & {{\mathrm{var}}\left( {\widehat {\bf{b}}_{{\mathrm{SBLUP}}_k}} \right)} \end{array}} \right]^{ - 1}} \hfill \\ {} \hfill & {} \hfill & {\left[ {\begin{array}{*{20}{c}} {{\mathrm{cov}}\left( {{\bf{b}}_{\it{f}},\widehat {\bf{b}}_{{\mathrm{SBLUP}}_1}} \right)} \\ \vdots \\ {cov\left( {{\bf{b}}_{\mathit{f}},\widehat {\bf{b}}_{{\mathrm{SBLUP}}_k}} \right)} \end{array}} \right]} \hfill \end{array}$$

Therefore, if **V**_SBLUP_ and **C**_SBLUP_ can be approximated then $\widehat {\bf{b}}_{{\mathrm{MT}} - {\mathrm{SBLUP}}}$ of Eq. (12) can be obtained from *k* single-trait $\widehat {\bf{b}}_{{\mathrm{SBLUP}}}$ estimates from Eq. (11).

To derive the approximations, we first consider the diagonal elements of **V**_SBLUP_, which comprise the variance of the SBLUP SNP solutions, ${\mathrm{var}}\left( {\widehat {\bf{b}}_{{\mathrm{SBLUP}}_k}} \right)$. These can be approximated from theory under the assumption that $\widehat {\bf{b}}_{{\mathrm{SBLUP}}_k}$ have BLUP properties ${\mathit{E}}\left[ {{\bf{b}}|{\hat{\bf b}}} \right] = {\hat{\bf b}}$, which in turn implies that ${\mathrm{cov}}\left( {{\bf{b}}_k,\widehat {\bf{b}}_{{\mathrm{SBLUP}}_k}} \right) = {\mathrm{var}}\left( {\widehat {\bf{b}}_{{\mathrm{SBLUP}}_k}} \right)$. Following Daetwyler et al.^37^ and Wray et al.^38^, the squared correlation between a phenotype, **y**_*k*_, in an independent sample and a single-trait BLUP predictor of the phenotype, $\widehat {\bf{g}}_{{\mathrm{BLUP}}_k}$, is approximately:

16 $$R_{{\bf{y}}_k,\widehat {\bf{g}}_{{\mathrm{BLUP}}_k}}^2 = R_k^2 \approx h_k^2{\mathrm{/}}\left( {1 + M_{{\rm eff}}\left( {1 - R_k^2} \right){\mathrm{/}}\left( {N_kh_k^2} \right)} \right)$$

where $\widehat {\bf{g}}_{{\mathrm{BLUP}}_k} = {\bf{W}}\widehat {\bf{b}}_{{\mathrm{BLUP}}_k}$ and $h_k^2$ is the proportion of phenotypic variance attributable to additive genetic effects for trait *k*. Note that *M*_eff_ is the effective number of chromosome segments or the number of independent SNPs, which is a function of effective population size (*N*_e_) and can be empirically obtained as an inverse of the variance of genomic relationships^39,40^. Here we use an estimate of *M*_eff_ of 60,000, which is in line both with our estimates from the genomic relationships in our simulation data and with previously reported estimates^41^. In Eq. (16), $R_k^2$ occurs on both the left- and righ-hand side. Solving for $R_k^2$ gives $R_k^2 = \frac{{\varphi + h^2 - \sqrt {\left( {\varphi + h^2} \right)^2 - 4\varphi h^4} }}{{2\varphi }}$, where *φ* is $\frac{{M_{{\mathrm{eff}}}}}{N}$.

With a phenotypic variance of 1 and individual-level genetic effects **g**_*k*_ = **Wb**_*k*_, then $h_k^2 = \sigma _{g_k}^2 = M\sigma _{b_k}^2$ and the squared correlation between the true, **g**_*k*_, and estimated BLUP effects, $\widehat {\bf{g}}_{{\mathrm{BLUP}}_k}$, is:

17 $$R_{{\bf{g}}_k,\widehat {\bf{g}}_{{\mathrm{BLUP}}_k}}^2 = R_k^2{\mathrm{/}}h_k^2$$

Rearranging Eq. (17) gives $R_k^2 = h_k^2R_{{\bf{g}}_k,\widehat {\bf{g}}_{{\mathrm{BLUP}}_k}}^2 = h_k^2\frac{{{\mathrm{cov}}\left( {{\bf{g}}_k,\widehat {\bf{g}}_{{\mathrm{BLUP}}_k}} \right)^2}}{{{\mathrm{var}}\left( {{\bf{g}}_k} \right){\mathrm{var}}\left( {\widehat {\bf{g}}_{{\mathrm{BLUP}}_k}} \right)}}$, which given the BLUP properties ${\mathrm{cov}}\left( {{\bf{g}}_k,\widehat {\bf{g}}_{{\mathrm{BLUP}}_k}} \right) = {\mathrm{var}}\left( {\widehat {\bf{g}}_{{\mathrm{BLUP}}_k}} \right)$ and $h_k^2 = \sigma _{g_k}^2$ with a phenotypic variance of 1, reduces to $R_k^2 = {\mathrm{cov}}\left( {{\bf{g}}_k,\widehat {\bf{g}}_{{\mathrm{BLUP}}_k}} \right)$ = ${\mathrm{var}}\left( {\widehat {\bf{g}}_{{\mathrm{BLUP}}_k}} \right) = M{\mathrm{var}}\left( {\widehat {\bf{b}}_{{\mathrm{BLUP}}_k}} \right)$. Therefore:

18 $${\mathrm{var}}\left( {\widehat {\bf{b}}_{{\mathrm{BLUP}}_k}} \right) = \frac{{{\mathrm{var}}\left( {\widehat {\bf{g}}_{{\mathrm{BLUP}}_k}} \right)}}{M} = \frac{{R_k^2}}{M}$$

Second, we consider the off-diagonal elements of **V**_SBLUP_, which are comprised of the covariance of BLUP SNP solutions among the *k* traits. These can again be approximated from theory given the covariance of genetic effects among traits *k* and *l* is cov(**b**_*k*_, **b**_*l*_) = *r*_G_*h*_*k*_*h*_*l*_/*M*, with *r*_G_ the genetic correlation, and given the squared correlation between the true genetic effects of the SNPs, **b**_*k*_, and $\widehat {\bf{b}}_{{\mathrm{BLUP}}_k}$ which is given by Eq. (17) as $R_{{\bf{b}}_k,\widehat {\bf{b}}_{{\mathrm{BLUP}}_k}}^2 = \frac{{R_k^2}}{M}{\mathrm{/}}\frac{{h_k^2}}{M} = R_k^2{\mathrm{/}}h_k^2$. The covariance of BLUP SNP predictors is then:

19 $${\mathrm{cov}}\left( {\widehat {\bf{b}}_{{\rm BLUP}_{k{\prime}}},\widehat {\bf{b}}_{{\rm BLUP}_l}} \right) = \frac{{R_k^2}}{{h_k^2}} \cdot \frac{{R_l^2}}{{h_l^2}}{\mathrm{cov}}\left( {{\bf{b}}_k,{\bf{b}}_{\mathit{l}}} \right) = \frac{{r_{\mathrm{G}}R_k^2R_l^2}}{{h_kh_lM}}$$

Finally, we can consider the column vector **C**_SBLUP_, which is composed of the covariance between the true genetic effects of the SNPs for the focal trait, **b**_*f*_, and $\widehat {\bf{b}}_{{\mathrm{SBLUP}}_k}$ for all of the *k* traits. The first element of **C**_SBLUP_ is covariance between the true genetic effects of the SNPs for the focal trait **b**_*f*_ and $\widehat {\bf{b}}_{{\mathrm{SBLUP}}_f}$ for the focal trait ${\mathrm{cov}}\left( {{\bf{b}}_f,\widehat {\bf{b}}_{{\mathrm{BLUP}}_f}} \right) = {\mathrm{var}}\left( {\widehat {\bf{b}}_{{\mathrm{BLUP}}_f}} \right) = \frac{{R_f^2}}{M}$. The remaining elements of **C**_SBLUP_ are ${\mathrm{cov}}\left( {{\bf{b}}_f,\widehat {\bf{b}}_{{\mathrm{BLUP}}_k}} \right)$, which can be approximated from theory by considering a regression of **b**_*f*_ on **b**_*k*_ where the regression coefficient $\beta _{f,k} = r_{\mathrm{G}}\sqrt {{\mathrm{var}}\left( {{\bf{b}}_f} \right){\mathrm{/}}{\mathrm{var}}\left( {{\bf{b}}_k} \right)}$. The covariance of **b**_*f*_ and $\widehat {\bf{b}}_{{\mathrm{BLUP}}_k}$ can then be written as:

20 $${\mathrm{cov}}\left( {{\bf{b}}_f,\widehat {\bf{b}}_{{\mathrm{BLUP}}_k}} \right) = {\mathrm{cov}}\left( {\beta _{f,k}{\bf{b}}_k,\widehat {\bf{b}}_{{\mathrm{BLUP}}_k}} \right) = r_{\mathrm{G}}\frac{{R_k^2}}{M} \cdot \frac{{h_f}}{{h_k}}$$

If we consider a two-trait example where the focal trait that we want to predict is matched to the first of the two traits, Eqs. (18), (19) and (20) combine as:

21 $${\bf{w}}_{{\mathrm{SBLUP}}} = {\bf{V}}_{{\mathrm{SBLUP}}}^{ - 1}{\bf{C}}_{{\mathrm{SBLUP}}} = \left[ {\begin{array}{*{20}{c}} {\frac{{R_1^2}}{M}} & {\frac{{r_{\mathrm{G}}R_1^2R_2^2}}{{h_1h_2M}}} \\ {\frac{{r_{\mathrm{G}}R_1^2R_2^2}}{{h_1h_2M}}} & {\frac{{R_2^2}}{M}} \end{array}} \right]^{ - 1}\left[ {\begin{array}{*{20}{c}} {\frac{{R_1^2}}{M}} \\ {r_{\mathrm{G}}\frac{{R_2^2}}{M} \cdot \frac{{h_1}}{{h_2}}} \end{array}} \right]$$

giving the index for the focal trait as: $\widehat {\bf{b}}_{{\mathrm{wMT}} - {\mathrm{SBLUP}}_f} = {\bf{w}}_1\widehat {\bf{b}}_{S{\mathrm{BLUP}}_f} + {\bf{w}}_2\widehat {\bf{b}}_{{\mathrm{SBLUP}}_2}$ with solutions for the index weights of:

22 $$\begin{array}{*{20}{l}} {{{w}}_{\mathrm f}} \hfill & = \hfill & {\left( {1 - \frac{{{{r}}_{\mathrm G}^2R_2^2}}{{h_2^2}}} \right){\mathrm{/}}\left( {1 - \frac{{{{r}}_{\mathrm G}^2R_{\mathrm f}^2R_2^2}}{{h_{\mathrm f}^2h_2^2M}}} \right)} \hfill \\ {} \hfill & = \hfill & {\left( {1 - {{r}}_{\mathrm G}^2R_{{\bf{b}}_2,\widehat {\bf{b}}_{{\mathrm{BLUP}}_2}}^2} \right){\mathrm{/}}\left( {1 - {{r}}_{\mathrm G}^2R_{{\bf{b}}_f,\widehat {\bf{b}}_{{\mathrm{BLUP}}_f}}^2R_{{\bf{b}}_2,\widehat {\bf{b}}_{{\mathrm{BLUP}}_2}}^2} \right),{\mathrm{and}}} \hfill \\ {{{w}}_2} \hfill & = \hfill & {\left( {{{r}}_{\mathrm G}/\left( {h_fh_2} \right)\left( {h_f^2 - R_1^2} \right)} \right){\mathrm{/}}\left( {1 - \frac{{{{r}}_{\mathrm G}^2R_f^2R_2^2}}{{h_f^2h_2^2M}}} \right)} \hfill \\ {} \hfill & = \hfill & {{{r}}_{\mathrm G}\left( {h_f{\mathrm{/}}h_2} \right)\left( {1 - R_{{\bf{b}}_f,\widehat {\bf{b}}_{{\mathrm{BLUP}}_f}}^2} \right){\mathrm{/}}\left( {1 - {{r}}_{\mathrm G}^2R_{{\bf{b}}_f,{\hat{\mathbf b}}_{{\mathrm{BLUP}}_f}}^2R_{{\bf{b}}_2,\widehat {\bf{b}}_{{\mathrm{BLUP}}_2}}^2} \right)} \hfill \end{array}$$

For traits with low power, $R_k^2$ is usually very small. In that case, **V**_SBLUP_ can be well approximated by a diagonal matrix with entries $\frac{{R_k^2}}{M}$. *w*_*f*_ will become 1 and *w*_*k*_ for all other traits will be $r_{{\mathrm{G}}_{f,k}}\frac{{h_f}}{{h_k}}$. It may appear surprising that traits with higher SNP heritability have smaller weights than traits with lower SNP heritability. This can be explained by the fact that the variance of each BLUP predictor $\left( {R_k^2} \right)$ is approximately proportional to $h_k^4N$ if *M*_eff_ is large, and thus a trait with higher SNP heritability will still have a larger contribution to the multi-trait predictor than a trait with lower SNP heritability.

Equation (17) implies $R_{{\bf{b}}_k,\widehat {\bf{b}}_{{\mathrm{BLUP}}_k}}^2 = R_{{\bf{g}}_k,\widehat {\bf{g}}_{{\mathrm{BLUP}}_k}}^2 = R_k^2{\mathrm{/}}h_k^2$ and thus the index weights of Eq. (15) can be applied equally to BLUP solutions for the SNP effects or BLUP predictors for individuals of each trait as described in the main text in Eq. (1) through (3). Both $r_{{\mathrm{G}}_{k,l}}$ and $h_k^2$ of Eq. (15) can be obtained from summary statistic data using LD score regression^22^ and therefore $\widehat {\bf{b}}_{{\mathrm{MT}} - {\mathrm{BLUP}}}$ effects of Eq. (10), which would traditionally require individual-level phenotype–genotype data for all traits, can be approximated accurately in a computationally efficient manner using only publically available GWAS summary statistic data and an independent genomic reference sample.

### Index weighted multi-trait OLS SNP effects

In the previous section, we have shown how $\widehat {\bf{b}}_{{\mathrm{SBLUP}}}$ estimates for multiple traits can be combined to yield more accurate $\widehat {\bf{b}}_{{\mathrm{wMT}} - {\mathrm{SBLUP}}}$ SNP effects, which can be turned into $\widehat {\bf{g}}_{{\mathrm{wMT}} - {\mathrm{SBLUP}}}$ individual predictors that approach $\widehat {\bf{g}}_{{\mathrm{MT}} - {\mathrm{BLUP}}}$ accuracy. However, using a similar weighting we can also combine $\widehat {\bf{b}}_{{\mathrm{OLS}}}$ estimates for multiple traits into $\widehat {\bf{b}}_{{\mathrm{wMT}} - {\mathrm{OLS}}}$.

For SNP *j* and focal trait *f*, we have $\widehat {\bf{b}}_{{\mathrm{OLS}}}$ values for *k* traits, and we wish to obtain the index weights, *w*_*j*,*k*_, for each $\widehat {\bf{b}}_{{\mathrm{OLS}}_{j,k}}$ effect as:

23 $$\widehat {\bf{b}}_{{\mathrm{wMT}} - {\mathrm{OLS}}_{j,f}} = \mathop {\sum }\limits_k w_{j,k}{\hat{\bf b}}_{{\mathrm{OLS}}_{j,k}} = {\bf{w}}_j\prime \widehat {\bf{b}}_{{\mathrm{OLS}}_j}$$

Just like before, the optimal weights can be derived as:${\bf{w}}_{{\mathrm{OLS}}} = {\bf{V}}_{{\mathrm{OLS}}}^{ - 1}{\bf{C}}_{{\mathrm{OLS}}}$, where **C**_OLS_ is now a *k* × 1 column vector of the covariances of the $\widehat {\bf{b}}_{{\mathrm{OLS}}_k}$ values of the *k* traits with the true genetic effects of the SNPs for the focal trait, and **V**_OLS_ is a *k* × *k* variance–covariance matrix of the $\widehat {\bf{b}}_{{\mathrm{OLS}}}$ effects:

24 $$\begin{array}{*{20}{l}} {{\bf{w}}_{{\mathrm{OLS}}}} \hfill & = \hfill & {{\bf{V}}_{{\mathrm{OLS}}}^{ - 1}{\bf{C}}_{{\mathrm{OLS}}}} \hfill \\ {} \hfill & = \hfill & {\left[ {\begin{array}{*{20}{c}} {{\mathrm{var}}\left( {\widehat {\bf{b}}_{{\mathrm{OLS}}_1}} \right)} & \cdots & {{\mathrm{cov}}\left( {\widehat {\bf{b}}_{{\mathrm{OLS}}_1},\widehat {\bf{b}}_{{\mathrm{OLS}}_k}} \right)} \\ \vdots & \ddots & \vdots \\ {{\mathrm{cov}}\left( {\widehat {\bf{b}}_{{\mathrm{OLS}}_k},\widehat {\bf{b}}_{{\mathrm{OLS}}_1}} \right)} & \cdots & {{\mathrm{var}}\left( {\widehat {\bf{b}}_{{\mathrm{OLS}}_k}} \right)} \end{array}} \right]^{ - 1}} \hfill \\ {} \hfill & {} \hfill & {\left[ {\begin{array}{*{20}{c}} {{\mathrm{cov}}\left( {{\bf{b}}_{\mathit{f}},\widehat {\bf{b}}_{{\mathrm{OLS}}_1}} \right)} \\ \vdots \\ {{\mathrm{cov}}\left( {{\bf{b}}_{\mathit{f}},\widehat {\bf{b}}_{{\mathrm{OLS}}_k}} \right)} \end{array}} \right]} \hfill \end{array}$$

The diagonal elements of **V**_OLS_ are:

25 $${\mathrm{var}}\left( {\widehat {\bf{b}}_{{\mathrm{OLS}}_k}} \right) = \frac{{h_k^2}}{M} + \frac{1}{{N_k}}$$

The off-diagonal elements for trait *k* and *l* are

26 $${\mathrm{cov}}\left( {\widehat {\bf{b}}_{{\mathrm{OLS}}_k},\widehat {\bf{b}}_{{\mathrm{OLS}}_l}} \right) = \frac{{r_{\mathrm{G}}h_kh_l}}{M}$$

**C**_OLS_ now has elements

27 $${\mathrm{cov}}\left( {{\bf{b}}_{\mathrm{k}},\widehat {\bf{b}}_{{\mathrm{OLS}}_k}} \right) = \frac{{r_{\mathrm{G}}h_kh_l}}{M}$$

If we again consider a two-trait example, Eqs. (25), (26) and (27) combine as:

28 $${\bf{w}}_{{\mathrm{OLS}}} = {\bf{V}}_{{\mathrm{OLS}}}^{ - 1}{\bf{C}}_{{\mathrm{OLS}}} = \left[ {\begin{array}{*{20}{c}} {\frac{{h_1^2}}{M} + \frac{1}{{N_1}}} & {\frac{{r_{\mathrm{G}}h_1h_2}}{M}} \\ {\frac{{r_{\mathrm{G}}h_1h_2}}{M}} & {\frac{{h_2^2}}{M} + \frac{1}{{N_2}}} \end{array}} \right]^{ - 1}\left[ {\begin{array}{*{20}{c}} {\frac{{h_1^2}}{M}} \\ {\frac{{r_{\mathrm{G}}h_1h_2}}{M}} \end{array}} \right]$$

These weights are considerably different from the BLUP weights, which reflects the different variances of BLUP effects and OLS effects. Here we include this section for completeness but focus our analyses on multi-trait BLUP effects, because they are more accurate in expectation than multi-trait OLS effects.

### Index weighted multi-trait SNP effects using LDPred

For phenotypes with a genetic architecture characterised by a few loci of very large effect sizes, this approach may not be ideal. Models that assume a mixture distribution for SNP effects, such as LDpred or BayesR, can yield higher prediction accuracies in traits of non-infinitesimal genetic architecture^18,42^. As outlined above, Eq. (17) implies $R_{{\bf{b}}_k,\widehat {\bf{b}}_{{\mathrm{BLUP}}_k}}^2 = R_{{\bf{g}}_k,\widehat {\bf{g}}_{{\mathrm{BLUP}}_k}}^2 = R_k^2{\mathrm{/}}h_k^2$ and thus the index weights of Eq. (15) can be applied equally to BLUP solutions for the SNP effects or BLUP predictors for individuals of each trait as described in the main text in Eq. (1) through (3). LDpred aims to estimate the posterior mean phenotype that provides best unbiased prediction. Therefore, single-trait individual-level predictors obtained from LDPred can also be weighted together to create an approximate multi-trait predictor.

### Prediction accuracy of weighted multi-trait BLUP predictors

The prediction accuracy of $\widehat {\bf{b}}_{{\mathrm{wMT}} - {\mathrm{BLUP}}}$ effects obtained from Eq. (15) can be derived by considering the correlation of **b**_*f*_ and $\widehat {\bf{b}}_{{\mathrm{wMT}} - {\mathrm{BLUP}}_k}$ as:

29 $$r_{{\bf{b}}_f,\widehat {\bf{b}}_{{\mathrm{wMT}} - {\mathrm{BLUP}}_f}} = \frac{{{\mathrm{cov}}\left( {{\bf{b}}_f,\widehat {\bf{b}}_{{\mathrm{wMT}} - {\mathrm{BLUP}}_f}} \right)}}{{\sqrt {{\mathrm{var}}\left( {\widehat {\bf{b}}_{{\mathrm{wMT}} - {\mathrm{BLUP}}_f}} \right){\mathrm{var}}\left( {{\bf{b}}_f} \right)} }}$$

Equation (13) gives $\widehat {\bf{b}}_{{\mathrm{wMT}} - {\mathrm{BLUP}}_f} = {{{\bf w}{\prime}}}\widehat {\bf{b}}_{{\mathrm{BLUP}}}$ and thus the covariance of **b**_*f*_ and $\widehat {\bf{b}}_{{\mathrm{wMT}} - {\mathrm{BLUP}}_f}$ is:

30 $${\mathrm{cov}}\left( {{\bf{b}}_f,\widehat {\bf{b}}_{{\mathrm{wMT}} - {\mathrm{BLUP}}_f}} \right) = {\mathrm{cov}}\left( {{\bf{b}}_f,{{{\bf w}{\prime}}}\widehat {\bf{b}}_{{\mathrm{BLUP}}}} \right) = {{{\bf w}{\prime}}}{\mathrm{cov}}\left( {{\bf{b}}_f,\widehat {\bf{b}}_{{\mathrm{BLUP}}}} \right) = {{{\bf w}{\prime}\bf C}}$$

The variance of the $\widehat {\bf{b}}_{{\mathrm{wMT}} - {\mathrm{BLUP}}}$ effects obtained from Eq. (15) is:

31 $${\mathrm{var}}\left( {\widehat {\bf{b}}_{{\mathrm{wMT}} - {\mathrm{BLUP}}}} \right) = {\mathrm{var}}\left( {{{{\bf w}{\prime}}}\widehat {\bf{b}}_{{\mathrm{BLUP}}_k}} \right) = {{{\bf w}{\prime}}}{\mathrm{var}}\left( {\widehat {\bf{b}}_{{\mathrm{BLUP}}_k}} \right){\bf{w}} = {{{\bf w}{\prime}\bf Vw}}$$

Additionally, **w** = **V**^−1^**C** and **Vw** = **C**, and thus **w**′**C** = **w**′**Vw** or written another way ${\mathrm{cov}}\left( {{\bf{b}}_f,\widehat {\bf{b}}_{{\mathrm{wMT}} - {\mathrm{BLUP}}_f}} \right) = {\mathrm{var}}\left( {\widehat {\bf{b}}_{{\mathrm{wMT}} - {\mathrm{BLUP}}}} \right)$ following BLUP properties. Substituting into Eq. (19), the correlation of **b**_*f*_ and $\widehat {\bf{b}}_{{\mathrm{wMT}} - {\mathrm{BLUP}}_k}$ can then be written as:

32 $$\begin{array}{*{20}{l}} {r_{{\bf{b}}_f,\widehat {\bf{b}}_{{\mathrm{wMT}} - {\mathrm{BLUP}}_f}}} \hfill & = \hfill & {{\mathrm{var}}\left( {\widehat {\bf{b}}_{{\mathrm{wMT}} - {\mathrm{BLUP}}}} \right){\mathrm{/}}\sqrt {{\mathrm{var}}\left( {\widehat {\bf{b}}_{{\mathrm{wMT}} - {\mathrm{BLUP}}}} \right){\mathrm{var}}\left( {{\bf{b}}_f} \right)} } \hfill \\ {} \hfill & = \hfill & {\sqrt {{\mathrm{var}}\left( {\widehat {\bf{b}}_{{\mathrm{wMT}} - {\mathrm{BLUP}}}} \right){\mathrm{/}}{\mathrm{var}}\left( {{\bf{b}}_f} \right)} } \hfill \end{array},$$

which gives the squared correlation as $R_{{\bf{b}}_f,\widehat {\bf{b}}_{{\mathrm{wMT}} - {\mathrm{BLUP}}_f}}^2$ = ${\mathrm{var}}\left( {\widehat {\bf{b}}_{{\mathrm{wMT}} - {\mathrm{BLUP}}}} \right){\mathrm{/}}{\mathrm{var}}\left( {{\bf{b}}_f} \right)$ = $\frac{{R_f^2}}{M}{\mathrm{/}}\frac{{h_k^2}}{M} = R_f^2{\mathrm{/}}h_k^2$. Therefore, the squared correlation between a phenotype and a multiple trait index weighted BLUP predictor of the phenotype is approximately:

33 $$R_{{\bf{y}}_k,\widehat {\bf{g}}_{{\mathrm{wMT}} - {\mathrm{BLUP}}_k}}^2 = M{\mathrm{var}}\left( {\widehat {\bf{b}}_{{\mathrm{wMT}} - {\mathrm{BLUP}}}} \right) = M{{{\bf w}{\prime}{\bf Vw}}}$$

If we consider a two-trait example then prediction accuracy for a focal trait $R_{{\bf{y}}_f,\widehat {\bf{g}}_{{\mathrm{wMT}} - {\mathrm{BLUP}}_k}}^2$ can be written as:

34 $$R_{{\bf{y}}_f,\widehat {\bf{g}}_{{\mathrm{wMT}} - {\mathrm{BLUP}}_f}}^2 = {\bf{w}}_f^2R_{{\bf{y}}_f,\widehat {\bf{g}}_{{\mathrm{BLUP}}_f}}^2 + {\bf{w}}_2^2R_{{\bf{y}}_2,\widehat {\bf{g}}_{{\mathrm{BLUP}}_2}}^2 + 2{\bf w}_f{\bf w}_2{\bf V}_{1,2}$$

where **V**_1,2_ is the off-diagonal element of the matrix **V** of Eqs. (15) and (21). The value of $R_{{\bf{y}}_f,\widehat {\bf{g}}_{{\mathrm{wMT}} - {\mathrm{BLUP}}_f}}^2$ can then be compared to the prediction accuracy of the single-trait BLUP predictor of Eq. (16) and to the prediction accuracy of a cross-trait predictor^43^, where a BLUP predictor of the second trait is used to predict the focal trait phenotype, which is given by: $R_{{\bf{y}}_f,\widehat {\bf{g}}_{{\mathrm{BLUP}}_2}}^2 = R_{{\bf{y}}_2,\widehat {\bf{g}}_{{\mathrm{BLUP}}_2}}^2r_{\mathrm{G}}\sqrt {\left( {h_2{\mathrm{/}}h_f} \right)}$. This comparison is of interest, because we expect the multi-trait predictor to be more accurate than any available single-trait predictor, even if the most accurate single-trait predictor is across two different traits. Cross-trait prediction is equivalent to the proxy-phenotype method, which has been used to predict cognitive performance from educational attainment GWAS data^44^.

### Loss of prediction accuracy from BLUP approximation

Equations (16), (17), (18), (19), (20), (21), (22), (23), (24), (25), (26), (27), (28), (29), (30), (31), (32), (33) and (34) assume that ${\mathrm{cov}}\left( {{\bf{b}}_k,\widehat {\bf{b}}_{{\mathrm{SBLUP}}_k}} \right)$ = ${\mathrm{var}}\left( {\widehat {\bf{b}}_{{\mathrm{SBLUP}}_k}} \right) = {\mathrm{var}}\left( {\widehat {\bf{b}}_{{\mathrm{BLUP}}_k}} \right)$, or in other words that SBLUP SNP solutions have BLUP properties. The use of an independent LD reference sample to create an approximate single-trait BLUP predictor in Eq. (11) does not affect the covariance between the true SNP effect sizes and the approximate BLUP SNP solution, meaning that the approximate single-trait BLUP predictors have BLUP properties. However, the variance of $\widehat {\bf{b}}_{{\mathrm{SBLUP}}}$ is likely affected, which may potentially result in a loss of prediction accuracy of a weighted multi-trait BLUP predictor. The variance of $\widehat {\bf{b}}_{{\mathrm{SBLUP}}}$ is:

35 $$\begin{array}{*{20}{l}} {\sigma _{\widehat {\bf{b}}_{{\mathrm{SBLUP}}}}^2} \hfill & = \hfill & {\left[ {\left[ {N{\bf{L}} + {\bf{I}}_M\lambda } \right]^{ - 1}{{{\bf W}{\prime}}}} \right]\left[ {{{{\bf W}{\prime}{\bf W}}}\sigma _{\mathit{b}}^2 + {\bf{I}}\sigma _{\mathit{e}}^2} \right]} \hfill \\ {} \hfill & {} \hfill & {\left[ {{\bf{W}}\left[ {N{\bf{L}} + {\bf{I}}_M\lambda } \right]^{ - 1}} \right]} \hfill \\ {} \hfill & = \hfill & {\left[ {N{\bf{L}} + {\bf{I}}_M\lambda } \right]^{ - 1}\left[ {\left( {{{{\bf W}{\prime}{\bf W}}}} \right)\left( {{{{\bf W}{\prime}{\bf W}}}} \right)\sigma _{\mathit{b}}^2} \right.} \hfill \\ {} \hfill & {} \hfill & {\left. { + {{{\bf W}{\prime}{\bf W}}}\sigma _{\mathit{e}}^2} \right]\left[ {N{\bf{L}} + {\bf{I}}_M\lambda } \right]^{ - 1}} \hfill \\ {} \hfill & = \hfill & {\left[ {\left( {\left[ {N{\bf{L}} + {\bf{I}}_M\lambda } \right]^{ - 1}\left( {{{{\bf W}{\prime}{\bf W}}}} \right)\left( {{{{\bf W}{\prime}{\bf W}}}} \right)\left[ {N{\bf{L}} + {\bf{I}}_M\lambda } \right]^{ - 1}} \right.} \right.} \hfill \\ {} \hfill & {} \hfill & {\left. {\left. { + \left[ {N{\bf{L}} + {\bf{I}}_M\lambda } \right]^{ - 1}{{{\bf W}{\prime}{\bf W}}}\lambda _k} \right)\left[ {N{\bf{L}} + {\bf{I}}_M\lambda } \right]^{ - 1}} \right]\sigma _{\mathit{b}}^2} \hfill \end{array}$$

The loss of information from using an independent data set as an LD reference to obtain **L**, rather than directly using the individual-level data to calculate **W**′**W**, can be approximated by considering the scenario where SNP makers are unlinked, resulting in diag[**L**]. The diagonal elements of $\sigma _{\widehat {\bf{b}}_{{\mathrm{SBLUP}}_{jj}}}^2$ for SNP *j* are then:

36 $$\sigma _{\widehat {\bf{b}}_{{\mathrm{SBLUP}}_{jj}}}^2 = \left( {\left[ {N + \lambda } \right]^{ - 2}{\mathrm{diag}}\left[ {\left( {{{{\bf W}{\prime}{\bf W}}}} \right)\left( {{{{\bf W}{\prime}{\bf W}}}} \right)} \right] + N\lambda \left[ {N + \lambda } \right]^{ - 2}} \right)\sigma _{\mathit{b}}^2$$

The diagonal elements of diag[(**W**′**W**)(**W**′**W**)] can be approximated as ${\mathrm{diag}}\left[ {\left( {{{{\bf W}{\prime}{\bf W}}}} \right)\left( {{{{\bf W}{\prime}{\bf W}}}} \right)} \right]$ ≈ $N^2\left( {1 + {\Bbb E}\left[ {r^2} \right]M} \right)$ + $N^2\left( {1 + M{\mathrm{/}}N} \right)$, where the expectation of the LD correlation of the SNPs, ${\Bbb E}\left[ {r^2} \right]$, is 1/*N* as the SNP markers are unlinked. Equation (36) can then be written as:

37 $$\begin{array}{*{20}{l}} {\sigma _{\widehat {\bf{b}}_{{\mathrm{SBLUP}}_{jj}}}^2} \hfill & = \hfill & {\left( {\left( {N^2 + NM + N\lambda } \right){\mathrm{/}}\left( {N + \lambda } \right)^2} \right)\sigma _{\mathit{b}}^2} \hfill \\ {} \hfill & = \hfill & {\sigma _{\mathit{b}}^2N{\mathrm{/}}(N + \lambda ) + \sigma _{\mathit{b}}^2NM{\mathrm{/}}(N + \lambda )^2} \hfill \end{array}$$

From Eq. (37), the squared correlation between true SNP effects and SBLUP SNP effects can be written as:

38 $$R_{{\bf{b}},\widehat {\bf{b}}_{{\mathrm{SBLUP}}}}^2 = N{\mathrm{/}}\left( {N + M + \lambda } \right) = N{\mathrm{/}}\left( {N + M{\mathrm{/}}h^2} \right)$$

This can be contrasted to Eq. (17), which gives the squared correlation between the true genetic effects of the SNPs, **b**_*k*_, and $\widehat {\bf{b}}_{{\mathrm{BLUP}}_k}$ as:

39 $$\begin{array}{*{20}{l}} {R_{{\bf{b}}_k,\widehat {\bf{b}}_{{\mathrm{BLUP}}_k}}^2} \hfill & = \hfill & {\frac{{R_k^2}}{M}{\mathrm{/}}\frac{{h_k^2}}{M} = R_k^2{\mathrm{/}}h_k^2} \hfill \\ {} \hfill & = \hfill & {1{\mathrm{/}}\left( {1 + M\left( {1 - R_k^2} \right){\mathrm{/}}\left( {N_kh_k^2} \right)} \right)} \hfill \\ {} \hfill & = \hfill & {N_k{\mathrm{/}}\left( {N_k + M\left( {1 - R_k^2} \right){\mathrm{/}}h_k^2} \right)} \hfill \end{array}$$

Equation (39) is similar to Eq. (38) apart from the factor $1 - R_k^2$. Therefore, the relative loss of prediction accuracy from using an SBLUP predictor is given as a ratio of Eqs. (39) and (38) as:

40 $$\frac{{R_{{\bf{b}},\widehat {\bf{b}}_{{\mathrm{SBLUP}}}}^2}}{{R_{{\bf{b}}_k,\widehat {\bf{b}}_{{\mathrm{BLUP}}_k}}^2}} = \frac{{Nh^2 + M}}{{Nh^2 + M\left( {1 - R_k^2} \right)}}$$

For a phenotype of SNP heritability 0.5, with effective number of independent markers (independent genomic segments), *M*_eff_, of ~ 60,000 and sample size, *N*, of 500,000, $R_{{\bf{b}},\widehat {\bf{b}}_{{\mathrm{SBLUP}}}}^2$ from summary statistics in an independent reference sample will be 91% of the value of $R_{{\bf{b}}_k,\widehat {\bf{b}}_{{\mathrm{BLUP}}_k}}^2$ if individual-level data were available. Likewise, for a two-trait example where both traits have *h*^2^ = 0.5 and *N* = 500,000, the accuracy of the multi-trait SBLUP predictor will also be 91% of the accuracy of the multi-trait BLUP predictor.

It should be noted that here we assume **L** to be a a diagonal matrix, which will lead to a conservative estimate of the accuracy of SBLUP relative to the accuracy of BLUP, and that this estimate is in fact equivalent to the expected accuracy of a polygenic risk predictor based on marginal OLS effects^28^. In practice, approximating **L** through an external reference data set leads to SBLUP predictors, which are more accurate than predictors based on marginal OLS effects but less accurate than predictors based on BLUP effects.

### Computation time

Computing weights and combing up to 930,000 SNP effects of 34 traits takes <10 min on an Intel Xeon E7-8837 processor with 2.76 GHz. Memory requirements do not extend much beyond the amount necessary to read in the summary statistics. Calculation of single-trait SBLUP effects is more computationally demanding, so we split the data by chromosome. Runtime for chromosome one was <40 min, with memory usage just under 10 GB.

### Simulation study

To compare the accuracy of single-trait and multi-trait genetic predictors created from SNP effects obtained from both individual-level and summary statistic data, we conducted a simulation study based on real genotypes from the Kaiser Permanente study (Genetic Epidemiology Research on Adult Health and Aging: GERA cohort) and simulated phenotypes.

From the GERA cohort, we selected 50,000 individuals of European ancestry (for definitions of European individuals and quality control (QC) of the genotypic data, see ref. ^45^). SNP QC procedures on the initial data sets consisted of per-SNP missing data rate of <0.01, minor allele frequency >0.01, per-person missing data rate <0.01 and Hardy–Weinberg disequilibrium *p*-value < 1 × 10^−6^. For the subsequent imputation, the data were first haplotyped using HAPI-UR. After that, Impute2 was used to impute the haplotypes to the 1000 genomes reference panel (release 1, version 3). Best-guess genotypes at common SNPs included in the HapMap 3 European sample were then extracted and filtered for imputation info score >0.5, missing data rate of <0.01, minor allele frequency >0.01, per-person missing data rate <0.01 and Hardy–Weinberg disequilibrium *p*-value < 1 × 10^−6^. Next, we performed principal component analysis and removed individuals with principal eigenvector values that were >7 SD from the mean of the European cluster. Lastly, we identified pairs of individuals with genetic relatedness matrix >0.05 and removed one individual from each of these pairs.

The Atherosclerosis Risk in Communities study (ARIC data) was used as an independent LD reference when estimating SBLUP SNP effects of Eq. (11). Eight thousand seven hundred and forty-four European individuals were selected and the data were imputed and QC conducted in the same way as described above for the GERA cohort. We then reduced the SNPs used in both the GERA and ARIC cohorts to overlapping HapMap3 SNPs, which gave 557,034 SNPs that were used in the simulation study.

We then randomly assigned 20,000, 20,000 and 10,000 individuals from the GERA cohort to create three data sets: training set one, training set two, and a testing set. We simulated two genetically correlated traits by randomly selecting 2000 causal SNPs. Effect sizes for the causal markers were simulated from a bivariate normal distribution with mean 0, variances of $\frac{{h_1^2}}{M}$ and $\frac{{h_2^2}}{M}$ and covariance of $r_{\mathrm{G}}\sqrt {h_1^2h_2^2}$. These effect sizes were then multiplied with the standardised genotype dosages (mean 0 and variance 1) to create a genetic value for each individual. Normally distributed environmental effects *e* ~ *N*(0, 1 − *h*^2^) were added to this genetic value for each individual to create phenotypes with mean 0 and variance of 1. To remove any effects of population stratification, the simulated phenotypes were then regressed against the first 20 genetic principal components, and the residuals from this regression were used in all subsequent analyses.

In training set 1, we simulated trait 1 and we then estimated: (i) OLS SNP effects using Eq. (5) $\left( {\widehat {\bf{b}}_{{\mathrm{OLS}}}} \right)$, (ii) BLUP SNP effects from the individual-level data using Eq. (8) $\left( {\widehat {\bf{b}}_{{\mathrm{BLUP}}}} \right)$, and (iii) approximate SBLUP effects using the OLS SNP effects from Eq. (5) and the ARIC data as a reference $\left( {\widehat {\bf{b}}_{{\mathrm{SBLUP}}}} \right)$. In training set 2, we simulated trait 2 and estimated $\widehat {\bf{b}}_{{\mathrm{OLS}}}$, $\widehat {\bf{b}}_{{\mathrm{BLUP}}}$ and $\widehat {\bf{b}}_{{\mathrm{SBLUP}}}$ in the same manner. We then estimated multi-trait BLUP SNP effects using Eq. (9) $\left( {\widehat {\bf{b}}_{{\mathrm{MT}} - {\mathrm{BLUP}}}} \right)$ from individual-level data by combining trait 1 from training set 1 and trait 2 from training set 2.

In the testing set, we then used the estimated SNP effects from the training sets to produce genetic predictors for both traits. Single-trait genetic predictors were created for both simulated traits from (i) the OLS SNP effects $\left( {\widehat {\bf{g}}_{{\mathrm{OLS}}}} \right)$, (ii) the BLUP SNP effects $\left( {\widehat {\bf{g}}_{{\mathrm{BLUP}}}} \right)$ and (iii) the SBLUP SNP effects $\left( {\widehat {\bf{g}}_{{\mathrm{SBLUP}}}} \right)$. We then created multi-trait predictors where trait 1 was the focal trait from: (i) individual-level multi-trait BLUP predictor $\left( {\widehat {\bf{g}}_{{\mathrm{MT}} - {\mathrm{BLUP}}}} \right)$, (ii) weighted multi-trait SBLUP predictor $\left( {\widehat {\bf{g}}_{{\mathrm{wMT}} - {\mathrm{SBLUP}}}} \right)$, (iii) a weighted multi-trait BLUP predictor-based individual-level single-trait BLUP estimates $\left( {\widehat {\bf{g}}_{{\mathrm{wMT}} - {\mathrm{BLUP}}}} \right)$, and (iv) a weighted multi-trait GWAS predictor based on GWAS OLS estimates $\left( {\widehat {\bf{g}}_{{\mathrm{wMT}} - {\mathrm{OLS}}}} \right)$. We simulated phenotypic values for both traits using the same effect sizes as those used to generate the phenotypes in the training sets and normally distributed environmental effects sampled independently for each trait as *e* ~ *N*(0, 1 − *h*^2^). We also compared estimates obtained using LDPred with the proportion of SNPs in the model of 0.001, 0.003, 0.01, 0.03, 0.1, 0.3, 1 or using the LDPred-Inf option.

We created two simulation scenarios. Heritability of the first and second trait and genetic correlations were $h_1^2$ = 0.2, $h_2^2$ = 0.8 and *r*_G_ = 0.8, respectively, in the first scenario and were $h_1^2$ = 0.5, $h_2^2$ = 0.5, and *r*_G_ = 0.5, respectively, in the second scenario. In each set-up, six replicates were conducted, each with a different set of randomly selected causal markers. We then repeated all analyses on a permuted data set, where the values of the genotype matrix were permuted across all individuals, for each SNP. This creates a genotype matrix where the allele frequency distribution remains the same, but all LD structure is removed, allowing us to determine the degree to which differences between the simulations results are driven by the LD structure in the real genotype data. Finally, because prediction accuracy is expected to be reduced by the error introduced by using an external LD reference data set and a restricted LD window when implementing Eq. (8) (see above), we examined how changing the LD reference and restricting the LD window size influences to optimal value of shrinkage parameter *λ* when implementing Eq. (8) (see Supplementary Fig. 3).

### Application to PGC schizophrenia and bipolar disorder

We then applied our approach to the schizophrenia (SCZ) and bipolar disorder (BIP) samples from both wave 1 and wave 2 data of the PGC (PGC1 and PGC2). A description of the data collection and imputation of the SNP genotype data can be found elsewhere^25,26,46^.

We selected these two disorders because there is a high genetic correlation between them (estimate for *r*_G_ between schizophrenia and bipolar disorder using ldsc: 0.72, SE: 0.03; estimated using meta-analysis of all PGC2 schizophrenia and bipolar cohorts, excluding cohorts which were used as test set in the initial PGC1 analysis), and it enabled us to draw a direct comparison between the approach described here and a previous study, which estimated multi-trait BLUP SNP effects $\left( {\widehat {\bf{b}}_{{\mathrm{MT}} - {\mathrm{BLUP}}}} \right)$ from individual-level data in an approach equivalent to Eq. (9). The previous study used PGC1 data in the training set and selected four cohorts for schizophrenia and three cohorts for bipolar disorder as test sets. For schizophrenia, the training set comprised 17 cohorts (8826 cases, 6106 controls) and for bipolar disorder the training set comprised 11 cohorts (5867 cases, 3328 controls). The test set of 4 cohorts for schizophrenia contained 4068 cases and 5471 controls, and the test set of 3 cohorts for bipolar disorder contained 2029 cases and 5338 controls. The analyses on the PGC1 data were performed on 745,705 HapMap3 SNPs in common across all data sets. To have a direct comparison to our previous study, we began by re-analysing the same PGC1 training set data to estimate: (i) OLS SNP effects using Eq. (5) $\left( {\widehat {\bf{b}}_{{\mathrm{OLS}}}} \right)$, (ii) BLUP SNP effects from the individual-level data using Eq. (8) $\left( {\widehat {\bf{b}}_{{\mathrm{BLUP}}}} \right)$, and (iii) approximate SBLUP effects using the OLS SNP effects from Eq. (5) and the ARIC data as a reference $\left( {\widehat {\bf{b}}_{{\mathrm{SBLUP}}}} \right)$ using Eq. (11). For the estimation of schizophrenia SBLUP effects, *λ* was set to 1,100,000, corresponding roughly to 1,000,000 markers and an observed scale SNP heritability estimate of 0.47, and for the estimation of bipolar disorder SBLUP effects, lambda was set to 1,200,000, corresponding roughly to 1,000,000 markers and an observed scale SNP heritability estimate of 0.45. For the four SCZ testing cohorts and the three BIP testing cohorts used in the previous study, we created: (i) weighted multi-trait SBLUP predictors $\left( {\widehat {\bf{g}}_{{\mathrm{wMT}} - {\mathrm{SBLUP}}}} \right)$, (ii) weighted multi-trait BLUP predictor-based individual-level single-trait BLUP estimates $\left( {\widehat {\bf{g}}_{{\mathrm{wMT}} - {\mathrm{BLUP}}}} \right)$, and (iii) weighted multi-trait GWAS predictor based on GWAS OLS estimates $\left( {\widehat {\bf{g}}_{{\mathrm{wMT}} - {\mathrm{OLS}}}} \right)$. We then compared the prediction accuracy we obtained using the weighted multi-trait SBLUP predictors to the individual-level multi-trait BLUP predictor $\left( {\widehat {\bf{g}}_{{\mathrm{MT}} - {\mathrm{BLUP}}}} \right)$ used in the previous study^16^.

We then extended our analysis to the PGC2 data set. There were 36 cohorts for schizophrenia (26,412 cases and 32,440 controls in total) and 23 cohorts for bipolar disorder (18,865 cases and 30,460 controls in total) available to us. The number of SNPs used in the PGC2 analyses varied between cohorts. Summary statistics for each of the PGC2 cohorts was available to an imputed SNP set of >10,000,000 SNPs. After intersecting this set of SNPs with the HapMap3 SNPs and the ARIC SNPs, 932,344 SNPs remained that were used to create predictors.

We applied a cross-validation approach as we observed that prediction accuracy as well as accuracy differences between predictors can be highly dependent on the choice of the test set in the extended PGC2 data set (Supplementary Figs. 5 and 6), which is supported by previous results showing highly variable prediction accuracy across cohorts in the PGC2 data set^25^. A cross-validation approach allowed us to get a more robust estimate of the increase of prediction accuracy achieved by our multi-trait prediction method compared to a single-trait predictor. We employed a leave-one-out cross-validation approach, where, for each test set cohort, all cohorts of the same disease without any highly related individuals were chosen to be in the training set for the single-trait predictor and all cohorts of both diseases without any highly related individuals were chosen to be in the training set for the multi-trait predictor. To identify pairs of cohorts with highly related individuals, genetic relatedness for all pairs of individuals (across all pairs of cohorts) was calculated based on chromosome 22, and whenever at least one pair of individuals had relatedness >0.8, that pair of cohorts was not simultaneously used in the training set and the test set.

The full genotypes from the PGC2 cohorts that were used as test sets underwent stringent QC and only comprised 458,744–860,576 SNPs for schizophrenia and 556,278–859,034 SNPs for bipolar disorder. We refrained from using the intersection between all these cohorts to not reduce the number of SNPs used in prediction by too much. This meant that different iterations in the cross-validations were based on predictions using a different number of SNPs. However, each comparison between a single-trait predictor and a multi-trait predictor is based on the same number of SNPs.

In each iteration of the cross-validation, a different cohort acts as the test set and a different set of cohorts comprises the training set. To create a predictor from a particular set of cohorts, we first had to obtain effect size estimates from this particular set of cohorts. This is achieved by performing a meta-analysis of the summary statistics of the cohorts that comprise the training set. The meta-analysed beta values *b*_META_ are calculated as:

41 $$b_{{\mathrm{META}}} = \frac{{\mathop {\sum }\nolimits_s \frac{{b_s}}{{{\mathrm{SE}}_s^2}}}}{{\mathop {\sum }\nolimits_s \frac{1}{{{\mathrm{SE}}_s^2}}}}$$

where *b*_*s*_ is the effect size in cohort *s* and SE_*s*_ is the standard error in cohort *s*. Conversion between beta values and odds ratios (OR) simply follows the equality *b* = log(OR). The weights derived for each trait make assumptions about the variance of SNP effects. We found that, in the summary statistics we used, the observed variance across SNP effects often departed from the expected value. To correct for that, we scaled the SNP effect estimates for each trait to have a variance of one and multiplied the weights for the unscaled SNP effects by the expected standard deviation across all SNPs.

We created approximate SBLUP effects $\left( {\widehat {\bf{b}}_{{\mathrm{SBLUP}}}} \right)$ using the OLS SNP effects from Eq. (5) and the ARIC data as an LD reference using Eq. (11) and set the shrinkage parameter, *λ*, to 1,300,000 for schizophrenia and to 2,000,000 for bipolar disorder, corresponding to observed scale SNP heritability estimates of 0.43 and 0.33 for schizophrenia and bipolar disorder, respectively. We then used the PLINK “--score” function to turn SNP effects $\left( {\widehat {\bf{b}}_{{\mathrm{SBLUP}}},\widehat {\bf{b}}_{{\mathrm{GWAS}}}} \right)$ into individual predictors $\left( {\widehat {\bf{g}}_{{\mathrm{SBLUP}}},\widehat {\bf{g}}_{{\mathrm{GWAS}}}} \right)$ for each meta-analysed schizophrenia or bipolar disorder cross-validation set. For the multi-trait weighting, we estimated the heritability of schizophrenia and bipolar disorder and their genetic correlation using LD score regression from publicly available PGC2 schizophrenia summary statistics and the PGC1 bipolar disorder summary statistics. These estimates were then used to calculate the index weights of Eq. (15) for the weighted multi-trait SBLUP predictors $\left( {\widehat {\bf{g}}_{{\mathrm{wMT}} - {\mathrm{SBLUP}}},\widehat {\bf{g}}_{{\mathrm{wMT}} - {\mathrm{GWAS}}}} \right)$ of SCZ and BIP, and these were not altered between different cross-validation sets.

To test the degree to which the choice of weights affects the accuracy of the multi-trait predictor, we compared the accuracy of multi-trait predictors based on a spectrum of other weights (Supplementary Figs. 5 and 6). For this, we took advantage of two things: First, when individual predictors $\left( {\widehat {\bf{g}}_{{\mathrm{SBLUP}}},\widehat {\bf{g}}_{{\mathrm{GWAS}}}} \right)$ are weighted rather than SNP effects $\left( {\widehat {\bf{b}}_{{\mathrm{SBLUP}}},\widehat {\bf{b}}_{{\mathrm{GWAS}}}} \right)$, the conversion from SNP effects to individual effects does not have to be repeated for different weights. Second, the scaling of a predictor does not influence its accuracy in terms of correlation between prediction and outcome. Therefore, rather than testing each combination of weights of schizophrenia and bipolar disorder, it is sufficient to vary the relative weight of schizophrenia to bipolar disorder to explore the whole range of possible multi-trait predictors for these two traits. For each test cohort, this enabled us to test whether the weights of our multi-trait predictor derived from theory deviate from the weights that would result in the highest prediction accuracy for that data set.

### Application to phenotypes in the UK Biobank study

We applied our approach to a large range of phenotypes for which GWAS summary statistics are publicly available. We started with GWAS summary statistics for 46 phenotypes. However, in some circumstance the same studies (i.e., based on the same individuals) had generated summary statistics for multiple similar phenotypes, so we chose only one phenotype per study, which left us with 34 phenotypes. For example, out of “Cigarettes per day” and “Smoking Ever” we only selected the latter to have only one trait for smoking. We used 112,338 unrelated individuals of European descent in the UK biobank data as the testing set. We paired 6 phenotypes out of the 34 summary statistic phenotypes to phenotypes in the UK Biobank: Height, BMI, fluid intelligence score, depression, angina, and diabetes. The first three are quantitative traits and the latter three are disease traits for which we could identify at least 1000 cases in the UK Biobank data. For details, see Supplementary Table 1.

For the disease traits, we used the self-reported diagnoses rather than ICD10 diagnoses, as they tend to have larger sample sizes. For depression, we used a more refined definition of cases and controls, where individuals were not counted as cases if they had any history of psychiatric symptoms or diagnoses other than depression or if they were prescribed drugs that are indicative of such diagnoses. Individuals were selected as controls only when there was an absence of any psychiatric symptoms or diagnoses and only when they were not prescribed any drugs that could be indicative of such diagnoses. All 6 traits in the UK Biobank were corrected for age, sex and the first 10 principal components by regressing the phenotype on these covariates and using the residuals from that regression for further analysis. For each trait, the SNPs that went into the analysis were based on the overlap between the GWAS summary statistics, the HapMap3 SNPs, the GERA data set, which was used as an LD reference in the SBLUP analysis, and the imputed SNPs from the UK Biobank. (For details on the QC process and imputation, see URLs.) Depending on the trait, the total number of SNPs ranged from around 660,000 to around 930,000.

We created single-trait $\left( {\widehat {\bf{g}}_{{\mathrm{SBLUP}}}} \right)$ as well as multi-trait $\left( {\widehat {\bf{g}}_{{\mathrm{wMT}} - {\mathrm{SBLUP}}}} \right)$ predictors for the six paired phenotypes. To create SBLUP SNP effects $\left( {\widehat {\bf{b}}_{{\mathrm{SBLUP}}}} \right)$ from summary statistic trait, we used a *λ* value of $M\left( {1 - h_{{\mathrm{SNP}}_k}^2} \right){\mathrm{/}}h_{{\mathrm{SNP}}_k}^2$ for each trait *k*, where *M* is assumed to be 1,000,000. As LD reference set, we used a random subset of 10,000 people of European descent from the GERA data set, and we set the LD window size to 2000 kb. We then used the PLINK “–score” function to turn SNP effects $\left( {\widehat {\bf{b}}_{{\mathrm{SBLUP}}}} \right)$ into individual predictors $\left( {\widehat {\bf{g}}_{{\mathrm{SBLUP}}}} \right)$ for each trait. For the multi-trait weighting, we used LD score regression to calculate SNP heritability and genetic correlation between all pairs of cohorts. For dichotomous disease traits, SNP heritability was calculated on the observed scale. For each phenotype for which a multi-trait predictor was created, we selected all phenotypes that had a genetic correlation estimate significantly different from 0 at *p* = 0.05 with the focal trait, as well as the focal trait itself. The summary statistics based single-trait SBLUP predictors of the selected phenotypes were then combined into multi-trait SBLUP $\left( {\widehat {\bf{g}}_{{\mathrm{wMT}} - {\mathrm{SBLUP}}}} \right)$ predictors. The weights for each phenotype were calculated according to Eq. (15). These weights require the single-trait predictors to have exactly the right variance. Since the summary statistics data slightly diverged from this expectation, we scaled each single-trait SBLUP predictor to have mean 0 and variance 1 and then multiplied it with its expected standard deviation, to ensure everything is on exactly the correct scale. We followed the same approach when using single-trait LDPred predictors.

We compared the performance of the multi-trait predictors $\left( {\widehat {\bf{g}}_{{\mathrm{wMT}} - {\mathrm{SBLUP}}}} \right)$ not only to the performance of the single-trait predictor $\left( {\widehat {\bf{g}}_{{\mathrm{SBLUP}}}} \right)$ for the same trait but also to the performance of all other (cross-trait) single-trait predictors for the traits that exhibited significant *r*_G_ with the focal trait (Fig. 4). This is appropriate because in some traits the single-trait predictor from the same trait is not the most accurate single-trait predictor.

### Code availability

Code reported in this manuscript is available from https://github.com/uqrmaie1/smtpred.

For GCTA, see http://cnsgenomics.com/software/gcta/

For LDSC, see https://github.com/bulik/ldsc

For MTG2, see https://sites.google.com/site/honglee0707/mtg2

For LDpred, see https://github.com/bvilhjal/ldpred/

For UK Biobank, see http://www.ukbiobank.ac.uk/

For PLINK2, see http://www.cog-genomics.org/plink2

### Data availability

PGC summary statistics data are available from http://www.med.unc.edu/pgc/results-and-downloads

For UK Biobank data, see https://www.ukbiobank.ac.uk/

## Electronic supplementary material

Supplementary Information

Descriptions of Additional Supplementary Files

Supplementary Data 1
