# Supplementary material for: Improving genetic prediction by leveraging genetic correlations among human diseases and traits
Source: Nat Commun. 2018 Mar 7;9:989. doi: 10.1038/s41467-017-02769-6 (PMC5841449; doi:10.1038/s41467-017-02769-6)
Supplement: Supplementary file 1 — Supplementary Information [file 41467_2017_2769_MOESM1_ESM.pdf]

Supplementary Figures

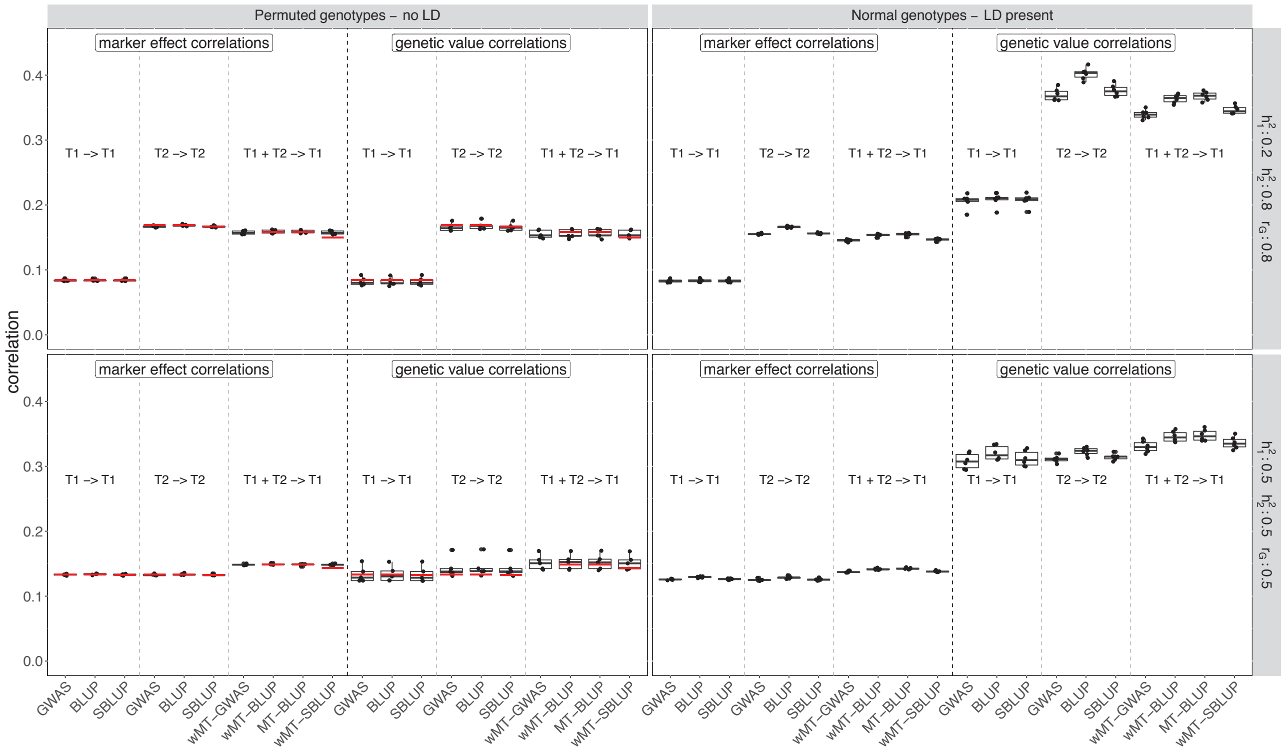

Supplementary Figure 1: Extended simulation results.

Each of the four panels has 10 predictors. For all predictors the correlation between simulated and estimated effect sizes are shown in the left half and the correlation between simulated genetic value and genetic predictor are shown in the right half. Predictors are ordered as follows: 1 to 3, predicting trait 1, while training on trait 1 (1: GWAS, 2: BLUP, 3: SBLUP); 4 to 6, predicting trait 2, while training on trait 2 (4: GWAS, 5: BLUP, 6: SBLUP); predicting trait 1, while training on trait 1 and 2 (multi trait predictor; 7: wMT-GWAS, 8: wMT-BLUP, 9: MT-BLUP, 10: wMT-SBLUP).

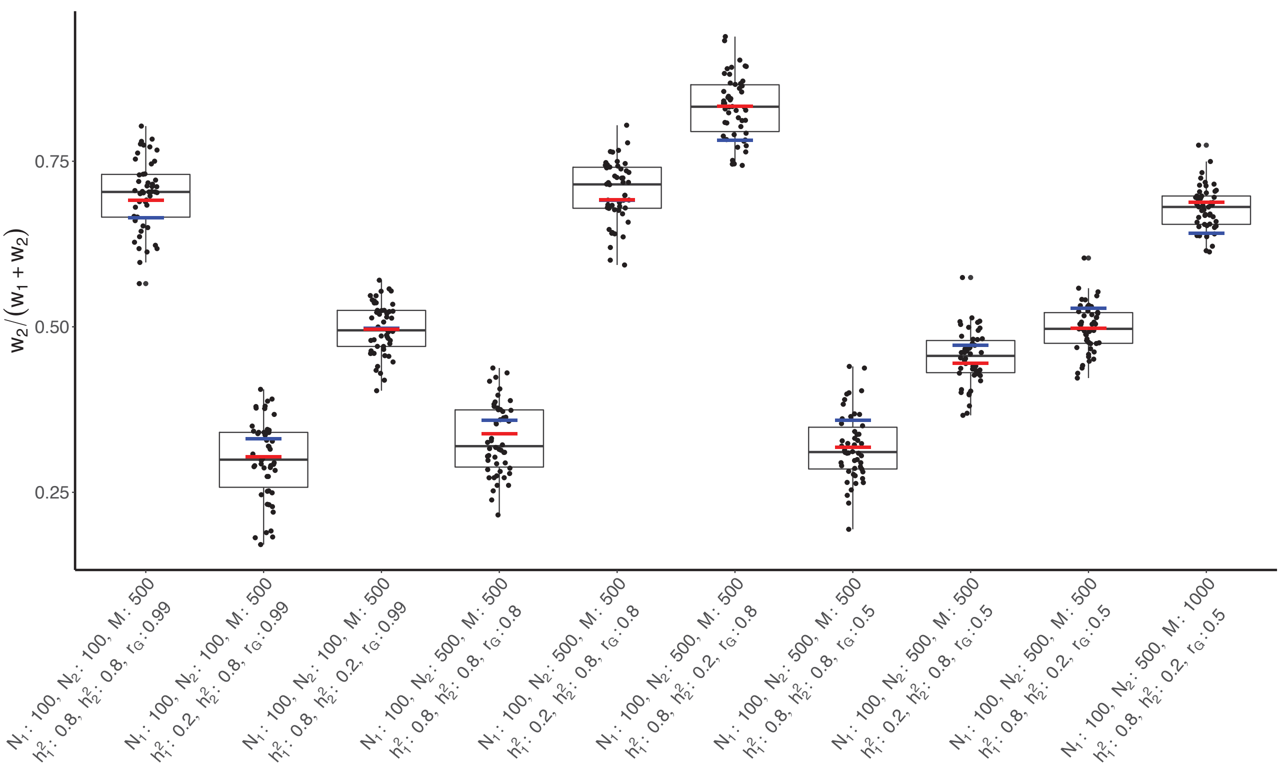

Supplementary Figure 2: Theoretically derived weights vs optimal weights in a small-scale simulation setup under a range of different parameters.

Two genetically correlated phenotypes were simulated under different values of h^2 (SNP heritability), r_G (genetic correlation), N (sample size) and M (number of markers). For each combination of parameters we calculated weights using Eq. [15] (red bars) and using the approximation $w_k = r_{G_{k,f}} \sqrt{h_k^2 N_k}$ for focal trait f and additional traits k, which assumes that the SNP effects of each trait k have equal variance (blue bars). For each parameter combination we replicated simulations 50 times and determined the weight ratio which resulted in the highest prediction accuracy (box plots and black dots). This confirms that the weights in Eq. [15] result in the highest prediction accuracy.

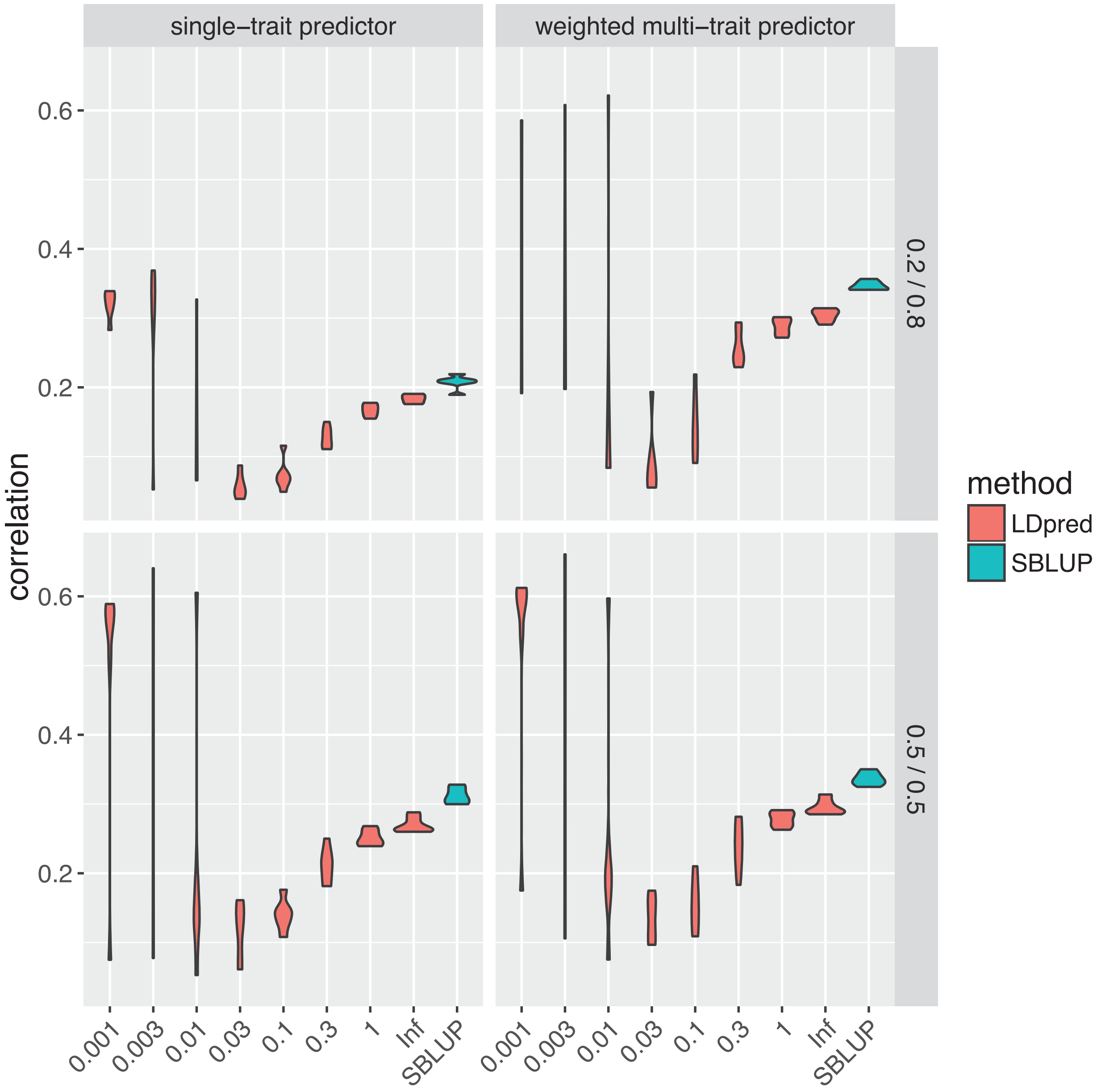

Supplementary Figure 3: Comparison of different methods for fitting linkage disequilibrium in simulations. SBLUP assumes an infinitesimal model, while the spike-and-slab model underlying LDpred is more general. Along the x-axis are different values for the LDpred parameter that specifies the fraction of causal markers. As our simulations are based on only 2000 causal markers, lower values for this parameter can lead to higher prediction accuracies. The SBLUP accuracy values shown here are the same as the SBLUP and wMT-SBLUP genetic value correlations with normal genotypes in Figure S1. In contrast to Figure S4, where the window size included all SNPs, window size here is restricted to 2000 kb for SBLUP or 333 SNPs for LDpred. Despite the approximate equivalence of these numbers, the window size restriction leads to a greater difference between the SNP effect estimates between SBLUP and LDpred-Inf than when window size is unrestricted for both methods.

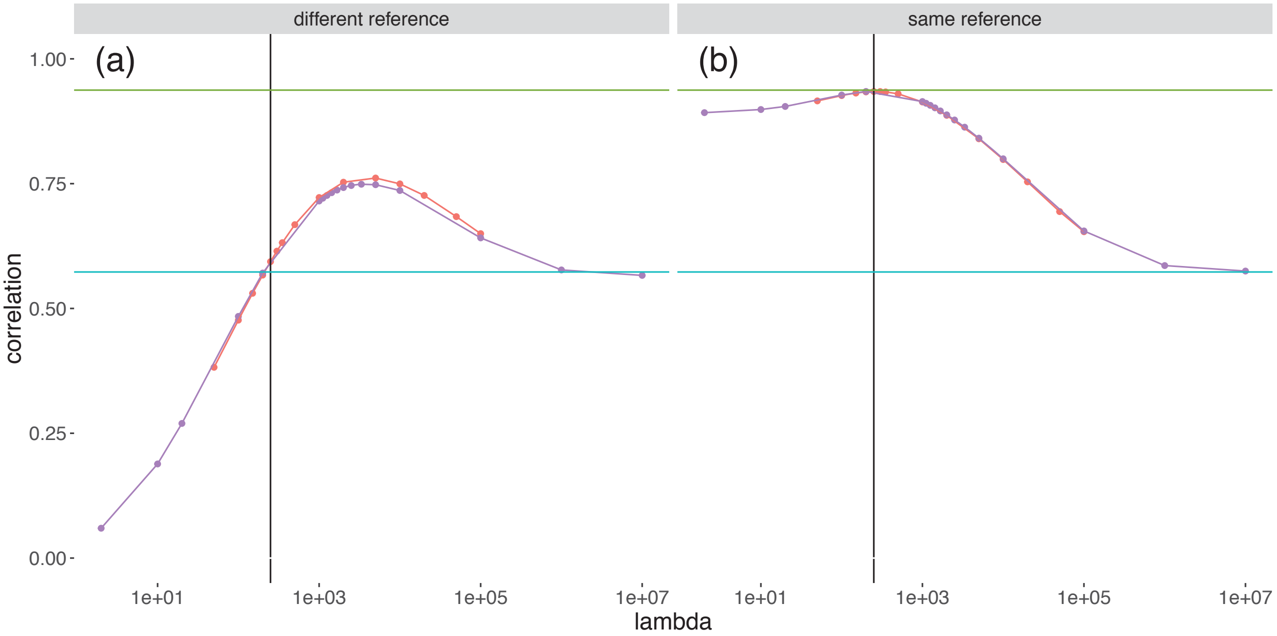

Supplementary Figure 4: Comparison of the accuracy of different methods to estimate simulated SNP effects.

Blue line: GWAS effect estimates (univariate OLS). Green line: BLUP estimates. Pink line: SBLUP estimates as a function of λ. Purple line: LDpred-inf estimates as a function of λ (where λ is calculated as $\frac{M}{h^2}$). Simulations are based on 20,000 individuals. 1000 SNPs from chromosome 22 were used so that the LD window included all SNPs. In (**a**) the external reference for SBLUP / LDpred-Inf is the same dataset as the genotypes which were used for simulation. Choosing an LD reference dataset which is different from the genotypes used for simulation (**b**) lowers prediction accuracy and increases the optimal value of λ.

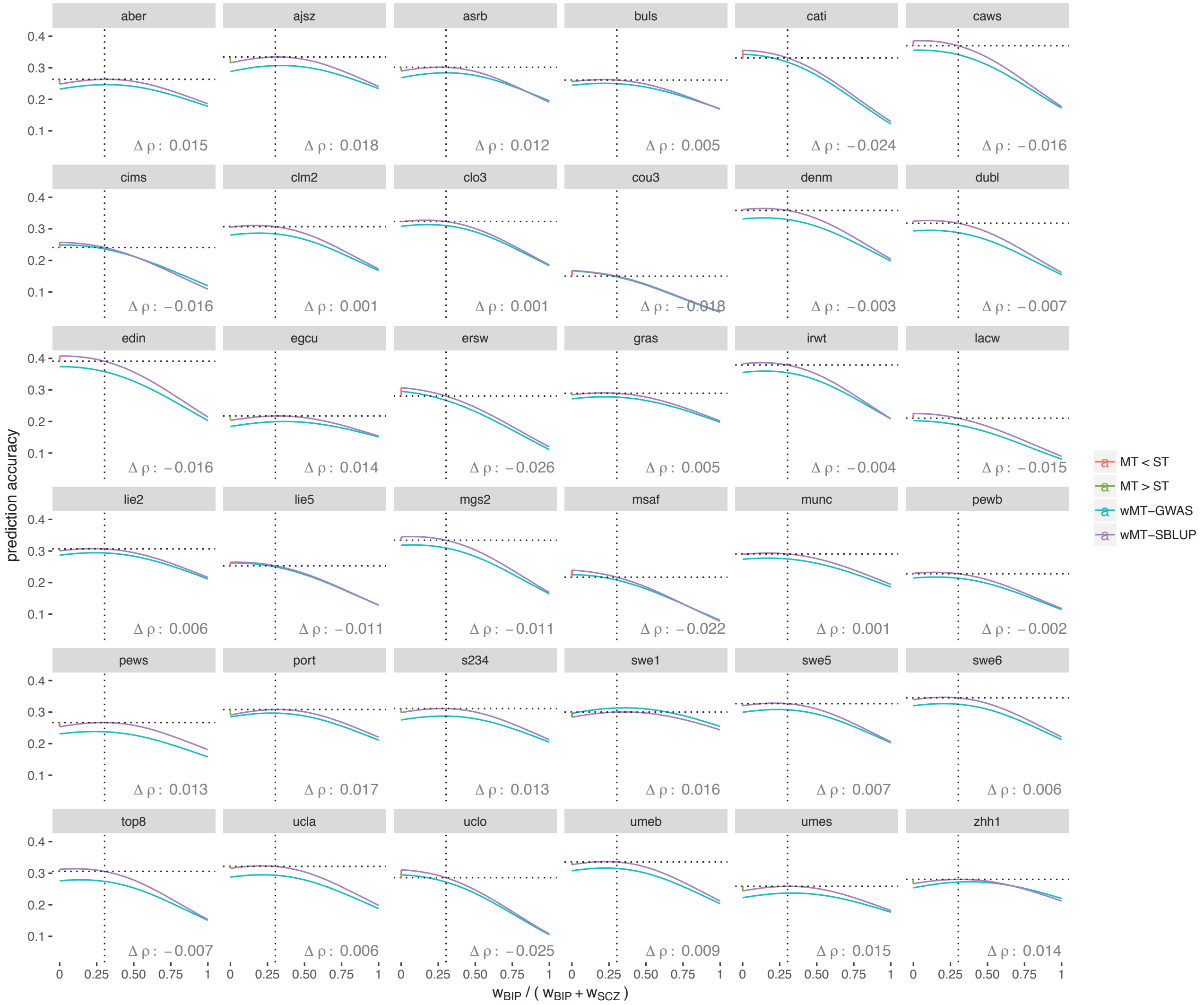

Supplementary Figure 5: Prediction accuracy for schizophrenia in each schizophrenia cohort using single-trait and multi-trait, GWAS (blue) and SBLUP (purple) predictors.

Each point along the x-axis represents a different multi-trait predictor with a different mixing proportion of schizophrenia and bipolar disorder data, corresponding to different weights. Dotted vertical lines indicate weights according to Eq. [15], and dotted horizontal lines indicate wMT-SBLUP prediction accuracy at these weights. If this prediction accuracy is higher than the single-trait prediction accuracy on the left hand side, the multi-trait predictor improves upon the single-trait predictor. The difference between single-trait and multi-trait accuracy ($\Delta\rho$) is visualized by green or red lines and printed in each panel.

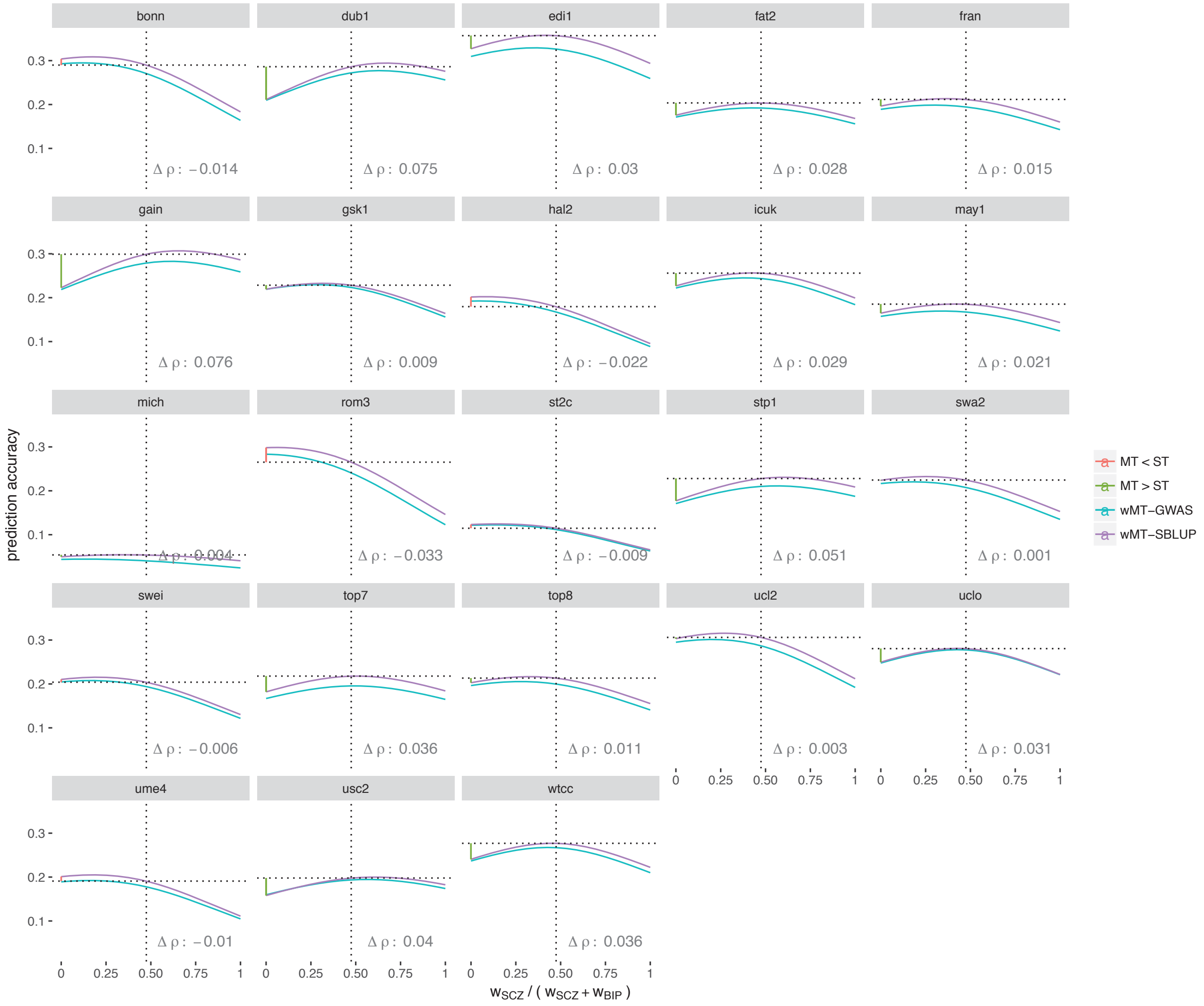

Supplementary Figure 6: Prediction accuracy for bipolar in each bipolar cohort using single-trait and multi-trait, GWAS (blue) and SBLUP (purple) predictors.

Each point along the x-axis represents a different multi-trait predictor with a different mixing proportion of schizophrenia and bipolar disorder data, corresponding to different weights. Dotted vertical lines indicate weights according to Eq. [15], and dotted horizontal lines indicate wMT-SBLUP prediction accuracy at these weights. If this prediction accuracy is higher than the single-trait prediction accuracy on the left hand side, the multi-trait predictor improves upon the single-trait predictor. The difference between single-trait and multi-trait accuracy ($\Delta\rho$) is visualized by green or red lines and printed in each panel.

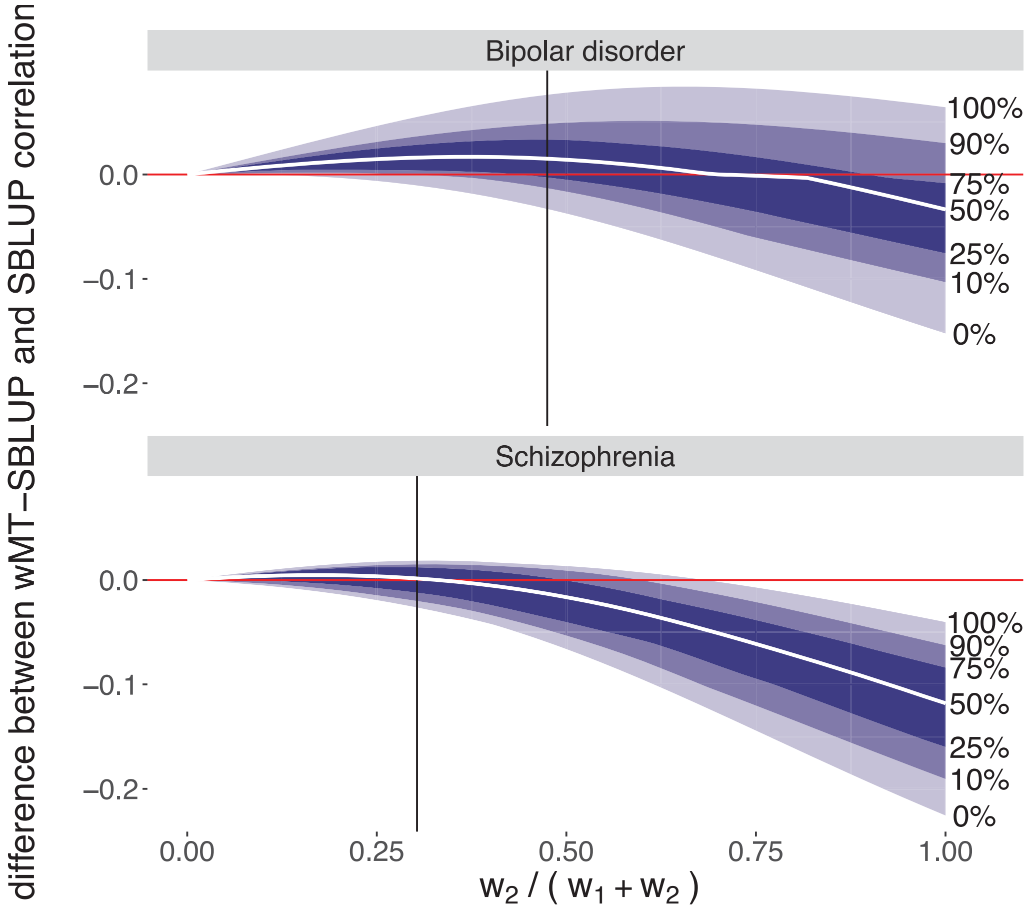

Supplementary Figure 7: Prediction accuracy difference between SBLUP predictors and wMT-SBLUP predictors, summarized over all cohorts.

A summary over all cohorts shown in **Supplementary Fig. S4** and **S5**. The y-axis now shows accuracy difference rather than absolute accuracy. For each weighting there is a distribution of prediction accuracy improvement (correlation single-trait predictor minus correlation multi-trait predictor) across all cross-validation iterations. The quantiles of this distribution are shown in shades of blue and the white line represents the median. The vertical line represents the weights derived from Eq. [15].

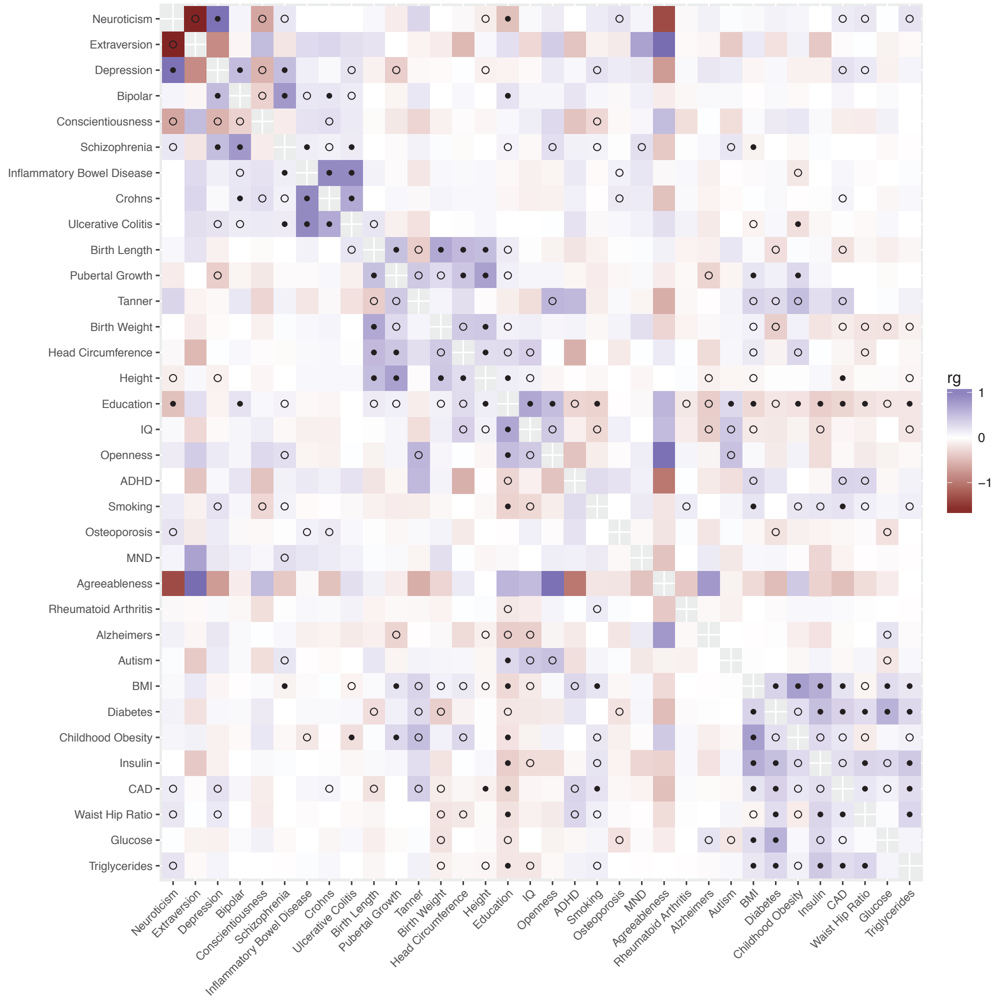

Supplementary Figure 8: Genetic correlation estimates between 34 traits.

LD score regression was used to estimate the genetic correlation based on summary statistics for each trait. Empty circles indicate a genetic correlation p-value lower than 0.05, filled circles indicate a genetic correlation p-value smaller than the Bonferroni threshold 0.05 / 561 = 8.91e-05. Traits are ordered according to hierarchical clustering based on the absolute value of genetic correlation estimates. Summary statistics were obtained from various sources, for details see **Supplementary Table S1**.

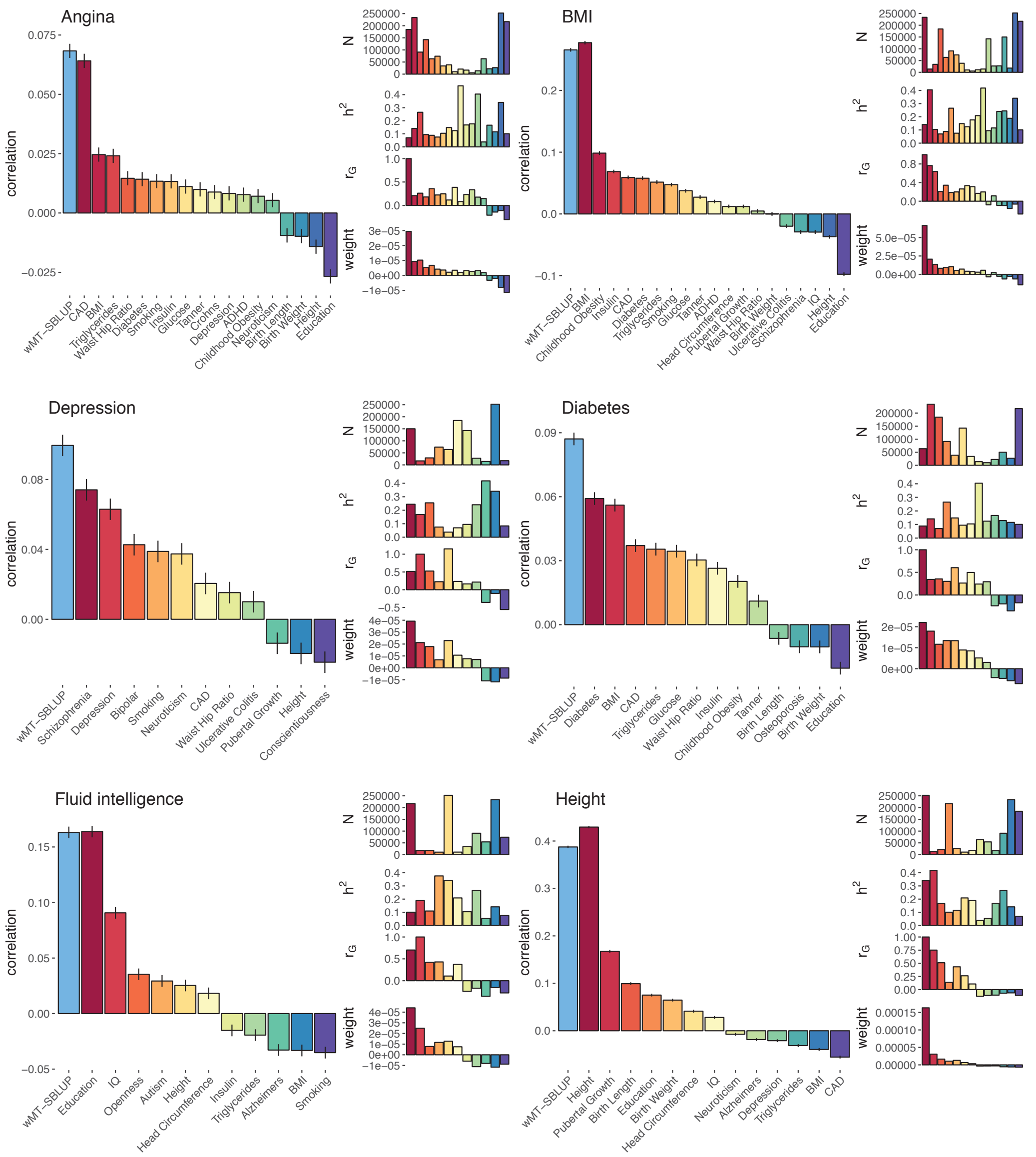

Supplementary Figure 9: Prediction accuracy for single-trait and multi-trait predictors in UK Biobank traits.

Prediction accuracy is shown as a correlation of genetic predictor and the phenotype for six traits in the UK Biobank for multi-trait predictors (light blue bars, wMT-OLS) and single-trait predictors (coloured bars show the SBLUP predictors for the traits as described in the x axis). Black bars show the correlation coefficient standard error. The multi-trait predictors for each trait are composed of all traits for which colourful bars are shown (r_G p-value < 0.05). Smaller bars on the right show, from top to bottom, sample size, SNP-heritability, r_G, and weights (given by Eq. [15]) for each trait.

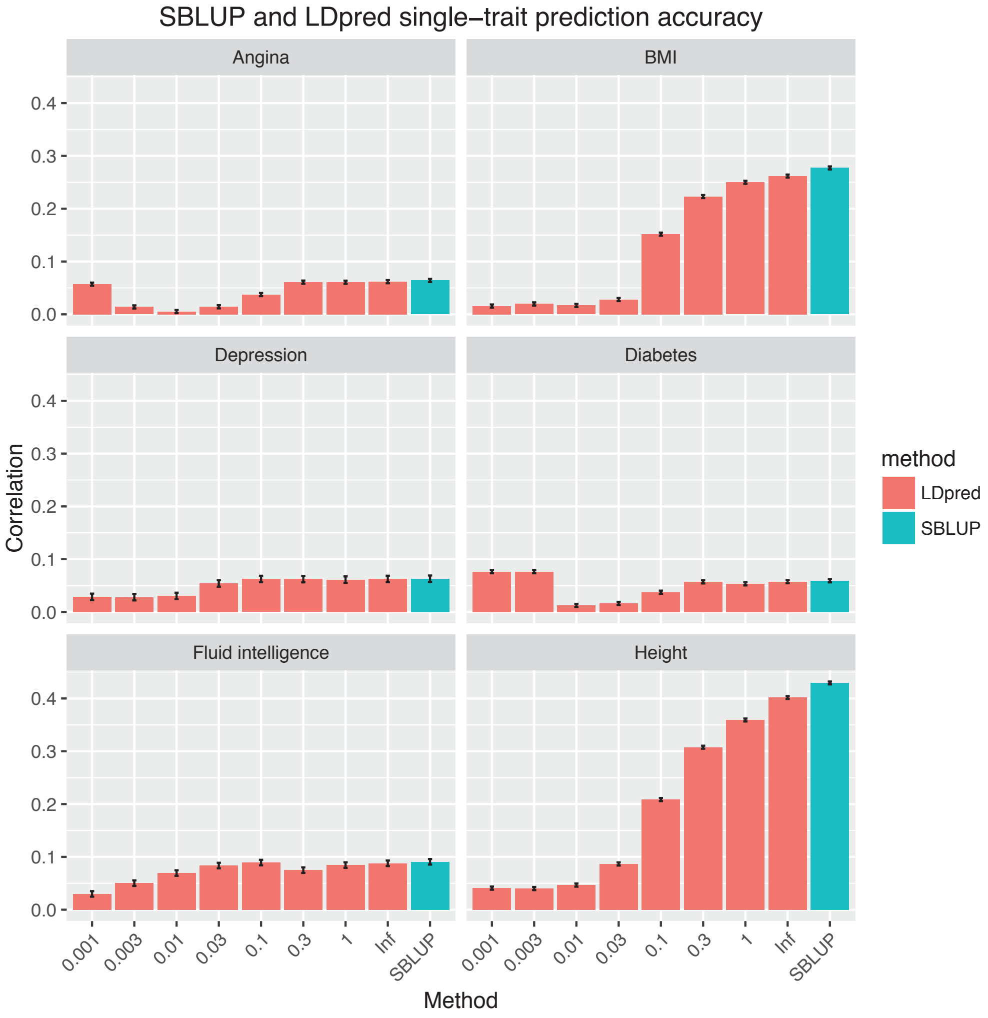

Supplementary Figure 10: Prediction accuracy for single-trait trait predictors in UK Biobank traits, using LDpred and using SBLUP to model LD.

Prediction accuracy for six traits in the UK Biobank for single-trait predictors generated from LDpred with different parameters for fraction of causal SNPs and generated from SBLUP. Only in diabetes does modelling a genetic architecture with fewer causal SNPs lead to a small improvement over SBLUP.

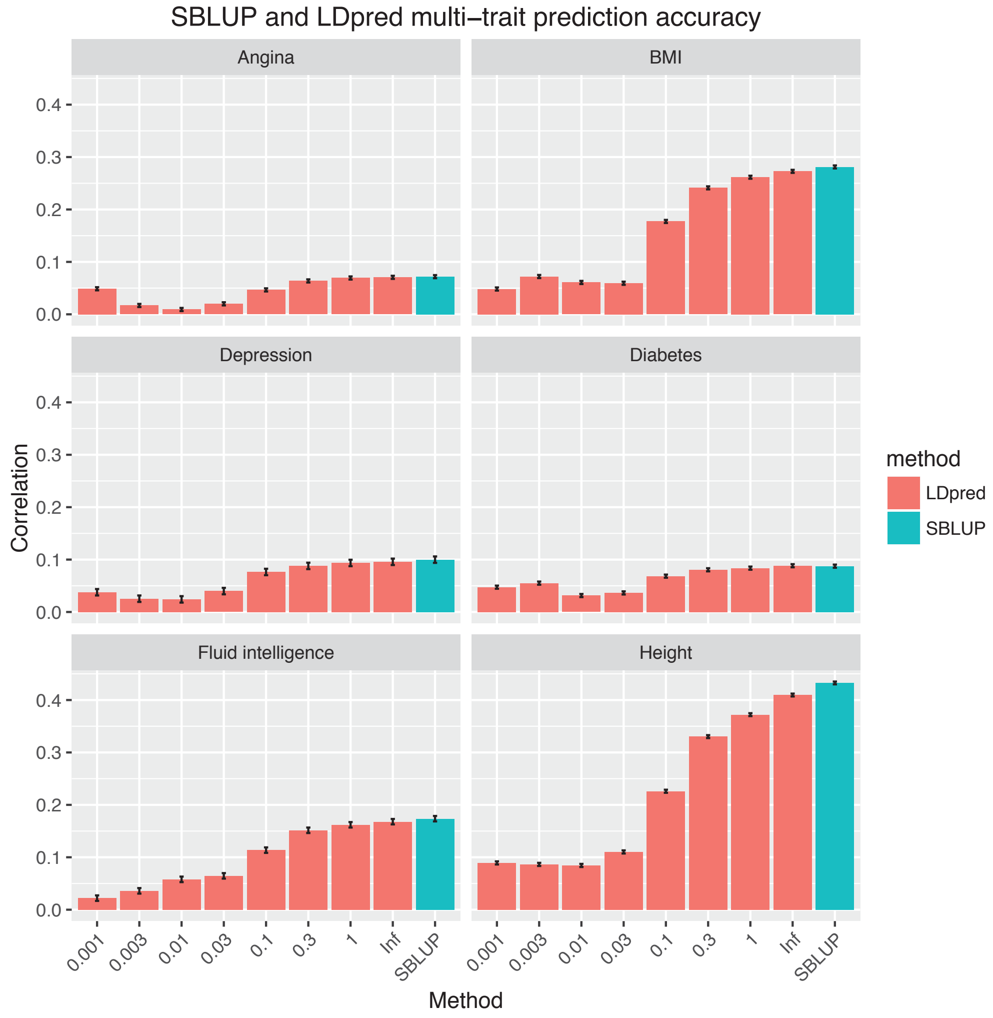

Supplementary Figure 11: Prediction accuracy for multi-trait trait predictors in UK Biobank traits, using LDpred and using SBLUP to model LD.

Prediction accuracy for six traits in the UK Biobank for multi-trait predictors generated from LDpred with different parameters for fraction of causal SNPs and generated from SBLUP. The patterns for the multi-trait prediction accuracies closely follow those of the single-trait prediciton accuracies for the different methods and paramters.

Supplementary Tables

Trait	h2	SE	Median N	PMID	UKB matched	UKB N
ADHD	0.17	0.1	5422	20732625		
BMI	0.14	0.01	233723	25673413	bmi	112027
Agreeableness	0.02	0.02	17375	21173776		
Alzheimers	0.05	0.03	54162	24162737		
Autism	0.41	0.05	10610	23453885		
Bipolar	0.27	0.02	29031	24280982	mania/bipolar disorder/ manic depression	301
Birth Length	0.17	0.02	22145	25281659		
Birth Weight	0.11	0.02	26836	23202124		
Childhood Obesity	0.42	0.05	13848	22484627		
Conscientiousness	0.08	0.03	17375	21173776		
CAD	0.07	0.01	184305	21378990	angina	3847
Crohn's disease	0.52	0.06	20883	26192919		
Education Years	0.11	0.01	216772	23722424		
Smoking	0.08	0.01	74035	20418890		
Extraversion	0.03	0.03	17375	21173776		
Glucose	0.15	0.03	38422	22581228		
Insulin	0.1	0.02	33823	22581228		
Head Circumference	0.21	0.05	10761	22504419		
Height	0.35	0.02	252190	20881960	height	112147
Inflammatory Bowel Disease	0.35	0.04	34652	26192919		
IQ	0.19	0.03	17989	23358156	fluid_intelligence _score	36093
Depression	0.18	0.03	16610	23453885	depression	4698
MND	0.04	0.02	36052	27455348		
Neuroticism	0.04	0.01	63661	25993607		
Openness	0.11	0.03	17375	21173776		
Osteoporosis Femur	0.13	0.02	49988	26367794		
Pubertal Growth	0.44	0.05	13955	23449627		
Rheumatoid Arthritis	0.23	0.06	25500	20453842		
Schizophrenia	0.25	0.01	150064	25056061	schizophrenia	131
Tanner	0.12	0.05	9915	24770850		
Triglycerides	0.27	0.06	90981	20686565		
Diabetes	0.09	0.01	63390	22885922	diabetes	4978
Ulcerative Colitis	0.27	0.04	27432	26192919		
Waist Hip Ratio	0.09	0.01	142471	25673412		

Supplementary Table 1: LD score regression h2 estimates, sample size and matched phenotypes

Supplementary Note 1

Further acknowledgements

ARIC

The Atherosclerosis Risk in Communities Study is carried out as a collaborative study supported by National Heart, Lung, and Blood Institute contracts (HHSN268201100005C, HHSN268201100006C, HHSN268201100007C, HHSN268201100008C, HHSN268201100009C, HHSN268201100010C, HHSN268201100011C, and HHSN268201100012C), R01HL087641, R01HL59367 and R01HL086694; National Human Genome Research Institute contract U01HG004402; and National Institutes of Health contract HHSN268200625226C. The authors thank the staff and participants of the ARIC study for their important contributions. Infrastructure was partly supported by Grant Number UL1RR025005, a component of the National Institutes of Health and NIH Roadmap for Medical Research.

GERA

The Genetic Epidemiology Research on Adult Health and Aging study was supported by grant RC2 AG036607 from the National Institutes of Health, grants from the Robert Wood Johnson Foundation, the Ellison Medical Foundation, the Wayne and Gladys Valley Foundation and Kaiser Permanente. The authors thank the Kaiser Permanente Medical Care Plan, Northern California Region (KPNC) members who have generously agreed to participate in the Kaiser Permanente Research Program on Genes, Environment and Health (RPGEH).

UK Biobank

This study has been conducted using UK Biobank resource under Application Number 12514. UK Biobank was established by the Wellcome Trust medical charity, Medical Research Council, Department of Health, Scottish Government and the Northwest Regional Development Agency. It has also had funding from the Welsh Assembly Government, British Heart Foundation and Diabetes UK

Genome-Wide Association Study of Schizophrenia (GAIN) and Molecular Genetics of Schizophrenia - nonGAIN Sample (MGS_nonGAIN)

Funding support for the companion studies, Genome-Wide Association Study of Schizophrenia (GAIN) and Molecular Genetics of Schizophrenia - nonGAIN Sample (MGS_nonGAIN), was provided by Genomics Research Branch at NIMH see below) and the genotyping and analysis of samples was provided through the Genetic Association Information Network (GAIN) and under the MGS U01s: MH79469 and MH79470. Assistance with data cleaning was provided by the National Center for Biotechnology Information. The MGS dataset(s) used for the analyses described in this manuscript were obtained from the database of Genotype and Phenotype (dbGaP) found at `http://www.ncbi.nlm.nih.gov/gap` through dbGaP accession numbers phs000021.v2.p1 (GAIN) and phs000167.v1.p1 (nonGAIN). Samples and associated phenotype data for the MGS GWAS study were collected under the following grants: NIMH Schizophrenia Genetics Initiative U01s: MH46276 (CR Cloninger), MH46289 (C Kaufmann), and MH46318 (MT Tsuang); and MGS Part 1 (MGS1) and Part 2 (MGS2) R01s: MH67257 (NG Buccola), MH59588 (BJ Mowry), MH59571 (PV Gejman), MH59565 (Robert Freedman), MH59587 (F Amin), MH60870 (WF Byerley), MH59566 (DW Black), MH59586 (JM Silverman), MH61675 (DF Levinson), and MH60879 (CR Cloninger). Further details of collection sites, individuals, and institutions may be found in data supplement Table 1 of Sanders et al. (2008; PMID: 18198266) and at the study dbGaP pages.

GAIN: Whole Genome Association Study of Bipolar Disorder

Funding support for the Whole Genome Association Study of Bipolar Disorder was provided by the National Institute of Mental Health (NIMH) and the genotyping of samples was provided through the Genetic Association Information Network (GAIN). The datasets used for the analyses described in this manuscript were obtained from the database of Genotypes and Phenotypes (dbGaP) found at http://www.ncbi.nlm.nih.gov/gap through dbGaP accession number phs000017.v3.p1. Samples and associated phenotype data for the Collaborative Genomic Study of Bipolar Disorder were provided by the The NIMH Genetics Initiative for Bipolar Disorder. Data and biomaterials were collected in four projects that participated in NIMH Bipolar Disorder Genetics Initiative. From 1991-98, the Principal Investigators and Co-Investigators were: Indiana University, Indianapolis, IN, U01 MH46282, John Nurnberger, M.D., Ph.D., Marvin Miller, M.D., and Elizabeth Bowman, M.D.; Washington University, St. Louis, MO, U01 MH46280, Theodore Reich, M.D., Allison Goate, Ph.D., and John Rice, Ph.D.; Johns Hopkins University, Baltimore, MD U01 MH46274, J. Raymond DePaulo, Jr., M.D., Sylvia Simpson, M.D., MPH, and Colin Stine, Ph.D.; NIMH Intramural Research Program, Clinical Neurogenetics Branch, Bethesda, MD, Elliot Gershon, M.D., Diane Kazuba, B.A., and Elizabeth Maxwell, M.S.W. Data and biomaterials were collected as part of ten projects that participated in the NIMH Bipolar Disorder Genetics Initiative. From 1999-03, the Principal Investigators and Co-Investigators were: Indiana University, Indianapolis, IN, R01 MH59545, John Nurnberger, M.D., Ph.D., Marvin J. Miller, M.D., Elizabeth S. Bowman, M.D., N. Leela Rau, M.D., P. Ryan Moe, M.D., Nalini Samavedy, M.D., Rif El-Mallakh, M.D. (at University of Louisville), Husseini Manji, M.D. (at Wayne State University), Debra A. Glitz, M.D. (at Wayne State University), Eric T. Meyer, M.S., Carrie Smiley, R.N., Tatiana Foroud, Ph.D., Leah Flury, M.S., Danielle M. Dick, Ph.D., Howard Edenberg, Ph.D.; Washington University, St. Louis, MO, R01 MH059534, John Rice, Ph.D, Theodore Reich, M.D., Allison Goate, Ph.D., Laura Bierut, M.D. ;

Johns Hopkins University, Baltimore, MD, R01 MH59533, Melvin McInnis M.D. , J. Raymond DePaulo, Jr., M.D., Dean F. MacKinnon, M.D., Francis M. Mondimore, M.D., James B. Potash, M.D., Peter P. Zandi, Ph.D, Dimitrios Avramopoulos, and Jennifer Payne; University of Pennsylvania, PA, R01 MH59553, Wade Berrettini M.D.,Ph.D. ; University of California at Irvine, CA, R01 MH60068, William Byerley M.D., and Mark Vawter M.D. ; University of Iowa, IA, R01 MH059548, William Coryell M.D. , and Raymond Crowe M.D. ; University of Chicago, IL, R01 MH59535, Elliot Gershon, M.D., Judith Badner Ph.D. , Francis McMahon M.D. , Chunyu Liu Ph.D., Alan Sanders M.D., Maria Caserta, Steven Dinwiddie M.D., Tu Nguyen, Donna Harakal; University of California at San Diego, CA, R01 MH59567, John Kelsoe, M.D., Rebecca McKinney, B.A.; Rush University, IL, R01 MH059556, William Scheftner M.D. , Howard M. Kravitz, D.O., M.P.H., Diana Marta, B.S., Annette Vaughn- Brown, MSN, RN, and Laurie Bederow, MA; NIMH Intramural Research Program, Bethesda, MD, 1Z01MH002810-01, Francis J. McMahon, M.D., Layla Kassem, PsyD, Sevilla Detera-Wadleigh, Ph.D, Lisa Austin,Ph.D, Dennis L. Murphy, M.D.
