# Supplementary material for: Improving genetic prediction by leveraging genetic correlations among human diseases and traits
Source: Nat Commun. 2018 Mar 7;9:989. doi: 10.1038/s41467-017-02769-6 (PMC5841449; doi:10.1038/s41467-017-02769-6)
Supplement: Supplementary file 2 — Descriptions of Additional Supplementary Files [file 41467_2017_2769_MOESM2_ESM.pdf]

Description of Supplementary Files

File Name: Supplementary Data 1
Description: LDSC rg estimates.
